# Prognostic evaluation of glycolysis markers in hepatocellular carcinoma: insights from meta-analysis and multi-omics approaches

**DOI:** 10.1186/s12920-025-02253-x

**Published:** 2025-11-08

**Authors:** Gangyi Li, Yongzhi Li, Jiale Zhou, Shuai Tang, Huaijuan Guo, Jie Lin

**Affiliations:** 1 Department of Ophthalmology, First People’s Hospital of Zigong, Zigong, 643000 Sichuan China; 2 Department of Hepatobiliary and Pancreatic Surgery, The Second Hospital of Jilin University, Changchun, 130000 China; 3 Department of Hepatobiliary and Pancreatic Surgery, the Neijiang First People’s Hospital, Neijiang, 641000 Sichuan Province China; 4 Department of Hepatobiliary and Pancreatic Surgery, Chengdu Medical College, Chengdu, 610500 Sichuan Province China; 5 Department of Pathology, Deyang People’s Hospital, Deyang, 618000 China; 6 Department of Oncology, the Affiliated Hospital of Yangzhou University, Yangzhou, 225000 China

**Keywords:** Hepatocellular carcinoma, Glycolysis gene set, Prognosis, Biomarker, Therapeutic target

## Abstract

**Background:**

Glycolysis, a central process of cellular energy metabolism, has been shown to be closely associated with the development of hepatocellular carcinoma (HCC). This study aimed to investigate the prognostic value of the glycolysis gene set (GGS) in HCC.

**Methods:**

Online databases were searched to identify studies on the correlation between glycolysis-related gene signature score and clinical characteristics in patients with HCC. HR and OR values with 95% CI were calculated. Bioinformatics analysis and in vitro validation were used to validate the results of the meta-analysis and investigate the potential oncogenic mechanisms of GGS.

**Results:**

Nineteen studies involving 3,406 patients were included. The pooled analysis showed that a high glycolysis-related gene signature score was associated with poor overall survival (OS) (HR = 1.98, 95% CI 1.59–2.46, *P* < 0.001), disease-free survival (DFS) (HR = 2.02, 95% CI 1.54–2.64, *P* < 0.001), and relapse-free survival (RFS) (HR = 2.38, 95% CI 1.39–4.08, *P* = 0.002). Bioinformatic and in vitro experiments confirmed the prognostic relevance and differential expression of GGS in HCC, and functional assays of *ENO1* further demonstrated its role in HCC progression.

**Conclusion:**

The upregulation of the glycolysis-related gene signature score is predominantly associated with poor prognosis in patients with HCC, suggesting that GGS may serve as a potential prognostic biomarker and therapeutic target for HCC, as exemplified by *ENO1* functional validation.

**Supplementary Information:**

The online version contains supplementary material available at 10.1186/s12920-025-02253-x.

## Introduction

Hepatocellular carcinoma (HCC) is the third deadliest cancer in the world [1]. Despite the numerous treatment options available for HCC, survival prognosis has not improved significantly, and treatment outcomes often vary among individuals [2]. Owing to the lack of early and effective diagnosis and the highly recurrent nature of HCC, there is an urgent need to identify promising prognostic biomarkers for clinical practice.

Glucose is degraded in cells through two main pathways: anaerobic conversion to lactate, which supplies a small amount of energy, and aerobic conversion, which supplies a large amount of energy. The small amount of energy produced during glycolysis is sufficient to maintain the physiological activity of normal cells. However, cancer cells, characterized by rapid proliferation and invasion, require more energy to meet their demands [3]. Glycolysis has been reported to be closely associated with the proliferation, invasion, immune escape, and survival prognosis of HCC [4]. A study demonstrated that the glycolysis gene set (GGS) is differentially expressed in tumors and paraneoplastic tissues and is commonly highly expressed in pan-cancers [5]. The relationship between glycolysis and cancer is known as the “Warburg effect” [6]. In addition to its direct involvement in the cellular energy supply, glycolysis affects the progression and therapeutic outcomes of HCC. Studies have demonstrated that glycolysis is modulated by non-coding RNAs and is closely correlated with the biological characteristics of HCC [7–9]. Additionally, research has shown that alterations in the glycolytic process can affect drug sensitivity in HCC [10, 11].

There are significant connections between GGS and HCC prognosis. Studies have reported that the overexpression of certain GGS, such as *PGK1* and *ALDOA*, in HCC is associated with cancer progression and poor prognosis [12–14]. However, other studies have held different views. High expression of certain GGS has been associated with longer disease-free survival (DFS) and overall survival (OS) in patients [15, 16]. The relationship between glycolytic biomarker expression and HCC prognosis remains controversial. The GGS was defined according to a previously published study that systematically characterized glycolysis genes across 24 cancer types [5]. We conducted analyses and validation on individual genes from this list, and candidate genes were further selected based on consistency with previous literature and the availability of clinical data. Ultimately, GGS (*ALDOA*, *ENO1*, *SLC2A1*, *PGK1*, *PKM*, and *TPI1*) were identified for downstream analyses. In contrast to prior studies that relied mainly on single-cohort bioinformatic analyses, our work integrates meta-analysis, bioinformatic validation, and experimental confirmation, thereby providing a more robust and evidence-based assessment of the prognostic value of glycolysis-related genes in HCC.

## Materials and methods

### Protocol and eligibility criteria

The systematic review and meta-analysis followed the standard guidelines provided by the Preferred Reporting Items for Systematic Reviews and Meta-Analyses (PRISMA) and was registered in the PROSPERO database (CRD42023418862). All the included studies focused on glycolysis-related gene signature scores in HCC and were published in English. All studies also included prognostic indicators such as OS, DFS, or relapse-free survival (RFS). Reviews, conference abstracts, letters, editorials, commentaries, case reports, and bioinformatic analyses were excluded. To avoid missing eligible literature, literature reference lists and conference abstracts/research reports in the supplementary issues were also carefully reviewed.

### Literature search

Up to May 1, 2025, a systematic search was carried out on studies related to GGS and HCC using databases including PubMed, Web of Science, and Embase. The search terms were (“GPI” OR “Glucose phosphate isomerase” OR “*ALDOA*” OR “Aldolase A” OR “TPI” OR “triosephosphate isomerase 1” OR “GAPD OR Glyceraldehyde-3-phosphate dehydrogenase” OR “*PGK1*” OR “Phosphoglycerate kinase 1” OR “*PGAM1*” OR “Phosphoglycerate mutase 1” OR “*ENO1*” OR “enolase 1” OR “*PKM*” OR “*PKM2*” OR “*PFKL*” OR “Phosphofructokinase” OR “*LDHA*” OR “Lactate dehydrogenase A” OR “*LDHB*” OR “Lactate dehydrogenase B” OR “OGDH OR Oxoglutarate” OR “glucose transporter type 1 (*GLUT1*)” OR “*SLC2A1*” OR “glucose transporter 1”) AND (“hepatocellular OR liver”) AND (“cancer” OR “tumor” OR “carcinoma” OR “adenocarcinoma” OR “neoplasia” OR “neoplasm”) AND (“prognosis” OR “prognostic” OR “prognoses” OR “survival” OR “outcome”). Additionally, we searched for potentially eligible literature via the reference lists of the included studies.

### Inclusion and exclusion criteria

The inclusion criteria were as follows: (1) pathological diagnosis of HCC; (2) measurement of glycolysis biomarkers using immunohistochemistry (IHC) or reverse transcription quantitative polymerase chain reaction (RT-qPCR); (3) studies describing glycolysis-related markers; and (4) studies describing the correlation between the expression of glycolysis biomarkers and survival outcomes, including OS, DFS, RFS, and their 95% confidence intervals (95% CI), or calculation of correlated HR values and their 95% CI from data available in the study. The exclusion criteria were as follows: (1) duplicate studies; (2) studies not based on humans; (3) reviews, case reports, meta-analyses, and clinical trials; (4) studies with no available valid data; and (5) studies with bioinformatics analysis or information downloaded from databases.

### Data extraction and quality evaluation

The information extracted from this study included the first author’s name, year of publication, region of publication, glycolysis biomarker name, total sample size (high or low expression level), follow-up time, glycolysis biomarker measurement methods, outcome indicators, HR values of survival outcomes and their 95% CI, and method of extraction of survival outcomes. We used the Newcastle-Ottawa scale (NOS) to score the included studies [17], where a study was considered to be of high quality with low publication bias if the score was no less than six points.

### Data download and sorting of the bioinformatics analysis

RNA sequencing data and clinical data for TCGA-LIHC were obtained from the TCGA database (https://portal.gdc.cancer.gov/repository), including 374 tumor samples and 50 paracancer samples. Next, we analyzed the transcript per million (TPM) expression of GGS in HCC, and ROC curves were used to assess the predictive value of GGS.

### Survival analysis and clinical correlation of GGS in HCC

We combined the results of the glycolysis-related gene signature score in HCC with the survival and clinical data. Kaplan-Meier plots were used to analyze the association of glycolysis-related gene signature scores (high and low expression by median expression value) with different survival prognoses of HCC. Finally, the correlation between the glycolysis-related gene signature score and clinicopathological parameters was evaluated using multifactor ROC curves and nomogram graphs.

### In vitro validation of the expression of the GGS gene in HCC lines

To bolster the reliability of our conclusions, we conducted in vitro experiments on the GGS gene following meta-analysis and bioinformatics validation at the transcriptomic level. We selected the normal human hepatocyte cell line (HL-7702) and HCC cell line (MHCC97H, Huh7, and HCC-LM3) to explore the expression profiles of these crucial GGS genes in different HCC strains. These cell lines were procured from the Cell Bank of the Chinese Academy of Sciences and cultured in a 5% CO_2_ incubator at 37 °C.

### Real-time quantitative PCR and western blot

PCR primer synthesis and other experimental consumables were procured from Sangon Biotech Co. Ltd. (Shanghai, China). The primer sequences are listed in Table S1. Total RNA was extracted using the TRIzol method, followed by cDNA synthesis using the Quanshijin reagent kit (Beijing Quanshijin Biotechnology Co., Ltd., China). The PCR reaction followed a two-step protocol provided by Bio-Rad (USA), and data were quantified using the accompanying software. Finally, data visualization was performed using the GraphPad Prism 9.5 software.

At the proteome level, western blot analysis of GGS was performed using the following steps. Proteins were extracted from the collected cells by RIPA buffer, analyzed by sodium dodecyl sulfate-polyacrylamide gel electrophoresis, and electrotransferred to a PVDF membrane. After blocking with 5% skim milk powder for one hour, the samples were incubated overnight at 4 °C with the following primary antibodies: anti-β-actin (ABclonal, AC026), anti-PKM (Proteintech, Cat. No: 10078-2-AP), anti-SLC2A1 (Proteintech, Cat. No: 21829-1-AP), anti-ENO1 (Proteintech, Cat. No: 11204-1-AP), anti-PGK1 (Proteintech, Cat. No: 17811-1-AP), anti-ALDOA (Proteintech, Cat. No. 11217-1-AP) and anti-TPI1 (Proteintech, Cat. No: 10713-1-AP). The membrane was subsequently incubated for one hour at room temperature with horseradish peroxidase-conjugated secondary antibodies (ab205719 or ab6721; Abcam). β-actin was used as an internal reference protein. Membranes were visualized and analyzed using an ECL chemiluminescent substrate and ECL imaging system. Finally, using the Human Protein Atlas (HPA) database, we obtained the immunohistochemistry data of GGS in normal and tumor tissues of HCC.

### Small interfering RNA transfection

Given the varying invasiveness of different HCC cell lines [18], we selected MHCC97H as the primary model for subsequent experiments. Using *ENO1* as an example, we further investigated the relationship between GGS and HCC progression. We used synthetic *ENO1* small interfering RNA (siRNA) to silence *ENO1* expression in intestinal cancer cells. SiRNAs were purchased from GenePharma Co., Ltd. (Suzhou, China). The RNA interference (RNAi) and negative control (NC) sequences were designed as follows: siENO1: 5’-GCAUUGGAGCAGAGGUUUAdTdT-3’(F), 5’-CCTGACTCAGTACAAGAAATT-3’(R); siNC: 5’-UUCUCCGAACGUGUCACGUTT-3’(F), 5’-ACGUGACACGUUCGGAGAATT-3’(R). MHCC97H cells were seeded in a 6-well plate at a density of 2 × 105 cells and incubated for 24 h. Once the cell density reached 60–70%, the siRNA was mixed with Lipofectamine 3000™ (Thermo Fisher Scientific, USA) and DMEM (Gibco, USA) and then incubated for 20 min. The mixture was added to the 6-well plate. The relative expression of *ENO1* was evaluated after 72 h of incubation using quantitative real-time PCR.

### MTT assay

Cell proliferation was detected using the MTT Cell Proliferation and Cytotoxicity Assay Kit (Beyotime, Nanjing, China). Briefly, cells were seeded at a density of 4 × 10³/well in 96-well plates 24 h after transfection. At different time points, 10 µL MTT (5 mg/mL) was added to each well, and cells were cultured for another 4 h. Furthermore, 100 µL of formazan solution was added into each well, and the optical density (OD) at 570 nm was measured on a microplate reader (PerkinElmer, Germany).

### Wound healing assay

After transfecting siENO1/siNC into MHCC97H, cells were collected from each group and seeded into a 6-well plate with 5 × 10^5^ cells per well. After incubation for a period of time, the cells were over 90% fused and were vertically scratched in a six-well plate using a 1 ml pipette tip. After scratching, the cells were washed with 1 × PBS to remove the separated cells. Then they were observed and photographed under a microscope to record the area of scratches at 0 and 48 h, respectively. The captured images were used to record the area between the two sides of the scratch, and software was used to analyze the cell migration area of each group of cells at 0 and 48 h.

### Transwell migration and invasion assays

The transwell migration and invasion experiments were conducted with an 8 μm/well Transwell chamber (Corning, USA). The microporous membrane in the upper chamber was covered with or without Matrigel (Corning, USA) for invasion and migration experiments. The chamber was placed into a 24-well plate, and 500 μm of DMEM medium containing 20% FBS was added into the lower chamber. Next, cells transfected with siRNA were collected from each group, and 2 × 105 cells were seeded into serum-free DMEM medium in the upper chamber. After 24 h of cultivation (migration assay) and 48 h (invasion assay), the chamber was fixed with 4% paraformaldehyde solution for 15 min and stained with 0.1% crystal violet staining solution at room temperature for 20 min. The cells in the upper layer of the microporous membrane were removed using cotton swabs. Five fields of view were randomly selected to count the number of perforated cells at 200 × magnification, and the values were taken for statistical analysis.

### Functional enrichment and immune analysis of GGS

Gene Ontology (GO) and Kyoto Encyclopedia of Genes and Genomes (KEGG) were used to analyze the functional enrichment of GGS. Next, the immune infiltration of HCC was assessed using ssGSEA to observe the immune cell composition (imc), immune cell function (imf) differences, and the relationship between GGS and immune checkpoints.

### Statistical analysis

Review Manager software (version 5.3) was used to pool HR and odds ratio (OR) values. I^2^ was used to assess the heterogeneity of the pooled results. In cases where I^2^ was greater than 50%, high heterogeneity was indicated and a random-effects model was used; when I^2^ was less than 50%, a fixed-effects model was used. For outcomes with significant heterogeneity, we further performed subgroup analyses and meta-regression to explore potential sources of heterogeneity. Sensitivity and publication bias analyses were performed using STATA 12.0. For continuous variables or dichotomous variables with fewer than 10 combined studies, we applied the Egger test to assess publication bias; for dichotomous variables with fewer than 10 combined studies, we used the Harbord test to assess publication bias [19]. If the P-value was greater than 0.05, there was no publication bias; otherwise, the trim-and-fill method was used to assess the reliability of the results. Statistical significance was set at less 0.05. Differential expression of GGS, its clinical correlation with HCC, and independent ROC curves were visualized using the limma, reshape2, ggplot2, ggpubr, and pROC packages. The survminer package was used to plot the GGS Kaplan-Meier plotter results in HCC. Immune infiltration analysis of HCC samples was carried out using the GSVA, GSEABase, Pacman, reshape2, psych, and ggcorrplotb packages. The final GGS functional enrichment analysis was completed using clusterProfiler, org.Hs.eg.db, enrichplot, circlize, RColorBrewer, and ComplexHeatmap packages with the screening criteria of PvalueFilter < 0.05 and QvalueFilter = 0.5.

## Results

### General characteristics of the included literatures

**Fig. 1:**
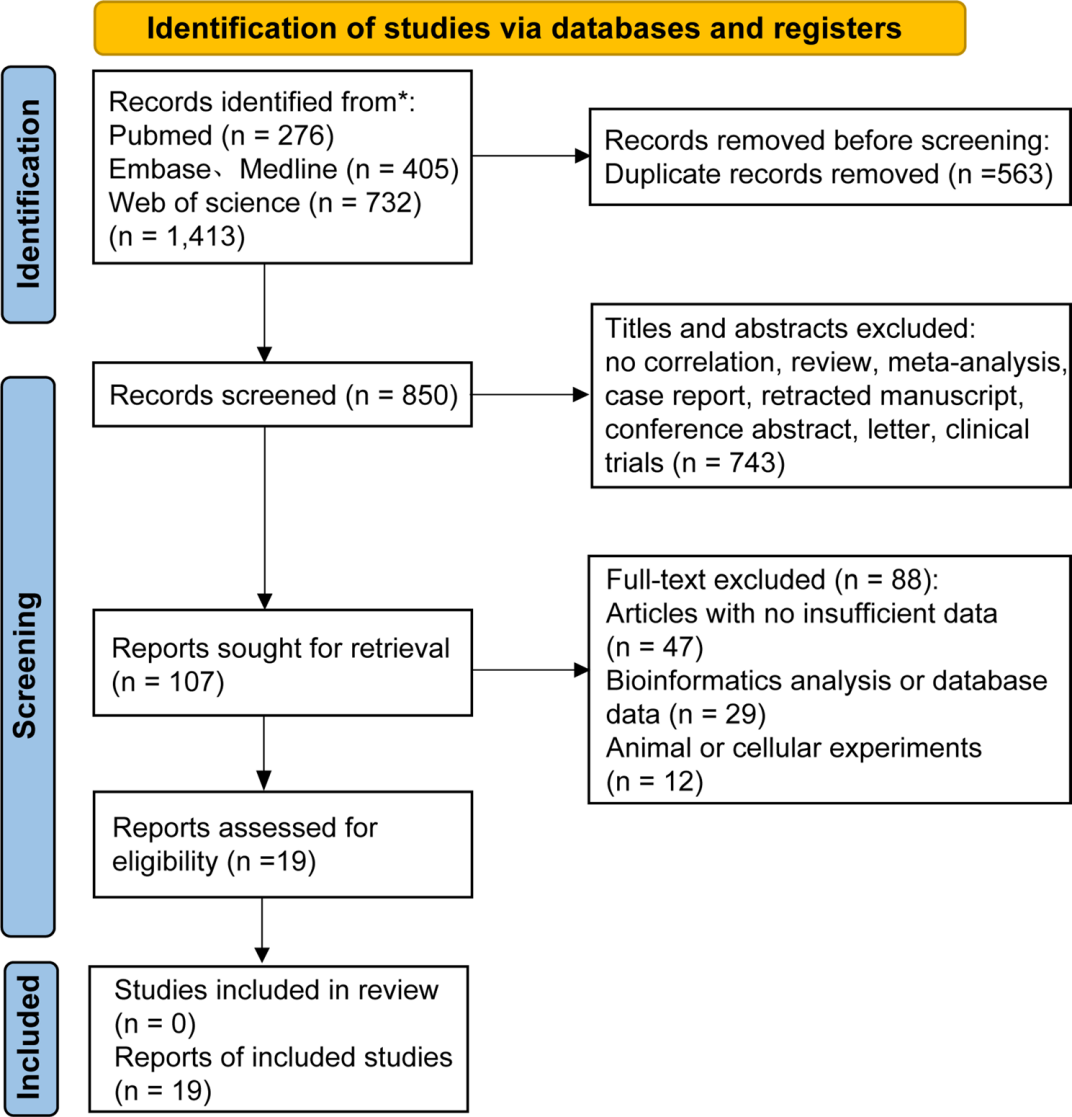
The PRISMA flow diagram of study selection and screening

Three databases were searched (PubMed = 276, Embase = 405, and Web of Science, 732), yielding 1,413 candidate studies. After removing duplicates, 850 unique studies were included. Subsequently, 743 studies were excluded due to irrelevant titles or abstracts. Ultimately, 19 studies were selected for meta-analysis based on the inclusion and exclusion criteria [13, 20–37] (Fig. 1). As summarized in Table 1, these studies assessed glycolysis-related gene signature score using quantitative reverse transcription polymerase chain reaction (RT-qPCR) and immunohistochemistry (IHC). Specifically, *PKM* (*n* = 11) [30–37], *SLC2A1* (*n* = 4) [24–27], *ALDOA* (*n* = 1) [20], *ENO1* (*n* = 3) [21–23], *PGK1* (*n* = 2) [13, 28], and *TPI1* (*n* = 1) [29]. For OS data, there were 18 unadjusted and 7 adjusted studies, while data on DFS and RFS were available from 5 to 4 studies, respectively. All included studies achieved scores >6 on the Newcastle-Ottawa Scale (NOS) (Table 2).

**Table 1:** Characteristics of included studies

Study	Region	Gene	expression type	Sample Size	Follow Up	Method	Outcome
				(High/Low)	(Month)		
Li 2019[20]	China	ALDOA	↑	100(53/47)	60	IHC	OS(UA), OS(MA), CP
Jiang 2020[21]	China	ENO1	↑	94(59/35)	100	IHC	OS(UA), CP
Zhang 2022[22]	China	ENO1	↑	135(65/70)	120	RT-qPCR	OS(UA), CP
Zhu 2018[23]	China	ENO1	↑	93(41/52)	84	IHC	OS(UA)
Chen 2018[24]	China	SLC2A1	↑	213(126/87)	120	IHC	OS(UA), CP
Shang 2020[25]	China	SLC2A1	↑	33(11/22)	50	RT-qPCR	RFS
Kitamura 2011[26]	Japan	SLC2A1	↑	63(23/40)	70	IHC	OS(UA), DFS
Sun 2016[27]	China	SLC2A1	↑	192(105/87)	96	IHC	OS(UA), CP, RFS
Hu 2017[13]	China	PGK1	↑	116(74/42)	100	IHC	OS(UA), PFS
Daskalow 2009[28]	Germany	PGK1	↑	60(37/23)	60	IHC	OS(UA)
Jiang 2017[29]	China	TPI1	↓	294(106/188)	60	IHC	OS(UA), CP
Chen 2015 −1[30]	China	PKM	↑	236(77/159)	80	RT-qPCR	OS(UA), OS(MA), CP
Chen 2015 −2[30]	China	PKM	↑	205(138/67)	80	RT-qPCR	OS(MA), CP
Hu 2015−1[31]	China	PKM	↑	490(256/234)	120	IHC	OS(UA), OS(MA), DFS, CP
Hu 2015−2[31]	China	PKM	↑	148(80/68)	120	IHC	OS(UA), OS(MA), DFS, CP
Li 2020[32]	China	PKM	↑	87(52/35)	100	IHC	OS(UA), DFS, CP
Liu 2015[33]	China	PKM	↑	367(89/278)	60	RT-qPCR	OS(UA), OS(MA), CP
Liu 2017−1[34]	China	PKM	↑	125(57/68)	100	IHC	RFS, CP
Liu 2017−2[34]	China	PKM	↑	97(30/64)	100	IHC	RFS, CP
Xu 2017[35]	China	PKM	↑	100(52/48)	60	IHC	OS(UA), DFS, CP
Zhao 2020[36]	China	PKM	↑	86(47/39)	36	IHC	OS(UA), OS(MA), CP
Zhou 2021[37]	China	PKM	↑	72(38/34)	50	IHC	OS(UA), CP

↑, upregulation; ↓, downregulation; IHC, immunohistochemistry; RT-qPCR, quantitative reverse transcription polymerase chain reaction; UA, univariate analysis; MA, multivariate analysis; CP, clinical parameters

**Table 2:** The risk of bias (NOS cohort)

Study	Domains			Results	
	Selection	Comparability	Outcome	Score	Risk
Li 2019[20]	****	**	**	8	Low
Jiang 2020[21]	****	**	**	8	Low
Zhang 2022[22]	****	**	**	8	Low
Zhu 2018[23]	****	*	**	7	Low
Chen 2018[24]	****	**	**	8	Low
Shang 2020[25]	****	*	*	6	Low
Kitamura 2011[26]	****	*	**	7	Low
Sun 2016[27]	****	**	**	8	Low
Hu 2017[13]	****	*	**	7	Low
Daskalow 2009[28]	****	*	**	7	Low
Jiang 2017[29]	****	**	**	8	Low
Chen 2015−1[30]	****	**	**	8	Low
Chen 2015−2[30]	****	**	**	8	Low
Hu 2015−1[31]	****	**	**	8	Low
Hu 2015−2[31]	****	**	**	8	Low
Li 2020[32]	****	**	**	8	Low
Liu 2015[33]	****	**	**	8	Low
Liu 2017−1[34]	****	**	**	8	Low
Liu 2017−2[34]	****	**	**	8	Low
Xu 2017[35]	****	**	**	8	Low
Zhao 2020[36]	****	**	*	7	Low
Zhou 2021[37]	****	**	*	7	Low

The NOS assessment contains nine stars divided into three categories: selection, comparability and outcome. Each star will be given score of 1 (**** = score of 4; ** = score of 2; * = score of 1). Score of ≥ 6 indicates the study is low risk of bias

### Prognostic significance of GGS in HCC patients of the meta-analysis

**Fig. 2:**
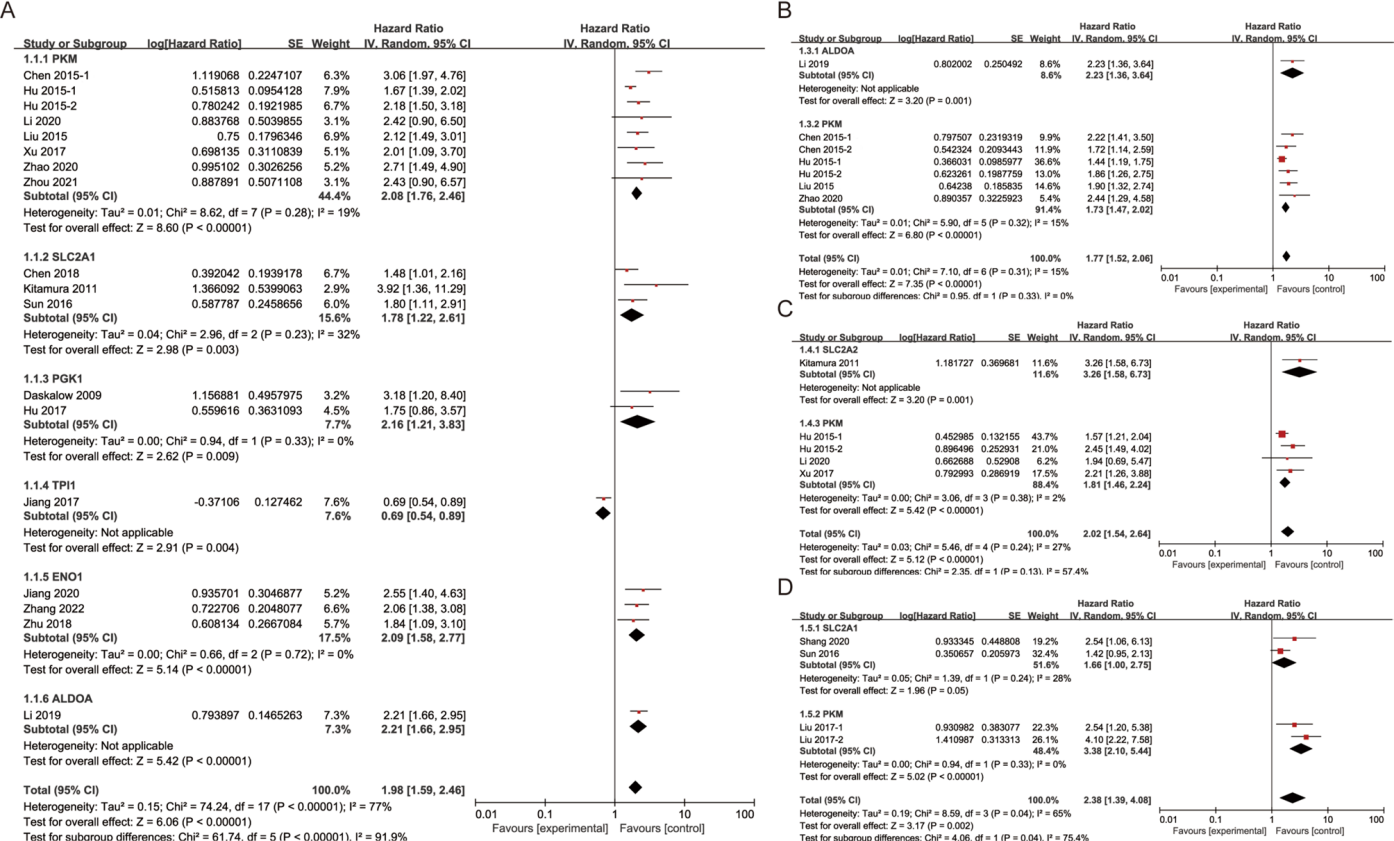
Prognostic significance of GGS in the HCC patients of the meta-analysis with random-effects model: (**A**) unadjusted OS. (**B**) adjusted OS. (**C**) DFS. (**D**) RFS

First, using a random-effects model (I^2^ = 77%, *P* < 0.001), a pooled analysis of the 18 unadjusted studies showed that partly high GGS was associated with worse OS in HCC (HR = 1.98, 95% CI 1.59–2.46, *P* < 0.001; Fig. 2A). In a subgroup analysis stratified by gene type, this correlation was significant in HCC patients with *PKM* (HR = 2.08, 95% CI 1.76–2.46, *P* < 0.001), *SLC2A1* (HR = 1.78, 95% CI 1.22–2.61, *P* = 0.003), *PGK1* (HR = 2.16, 95% CI 1.21–3.83, *P* = 0.009), *ENO1* (HR = 2.09, 95% CI 1.58–2.77, *P* < 0.001), and *ALDOA* (HR = 2.21, 95% CI 1.66–2.95, *P* < 0.001). Conversely, the correlation was inverse in patients with HCC with *TPI1* (HR = 0.69, 95% CI 0.54–0.89; *P* = 0.004). Seven adjusted studies indicated that upregulation of GGS was associated with poorer OS (HR = 1.77, 95% CI 1.52–2.06, *P* < 0.001, Fig. 2B). Specifically, patients with high expression of *ALDOA* (HR = 2.23, 95% CI 1.36–3.64, *P* = 0.001) and high expression of *PKM* (HR = 1.73, 95% CI 1.47–2.02, *P* < 0.001) had significantly shorter OS. To explore the sources of heterogeneity, we conducted a meta-regression analysis including four variables: gene, gene expression type, gene extraction method, and data extraction method (Figure S1 A-D, Table 3). The results indicated that gene expression type was the primary source of heterogeneity. To further explore this issue, we performed a subgroup analysis based on gene expression type (Figure S1 E), stratifying the studies into upregulated and downregulated gene groups. Notably, the downregulated group included only one study, whereas the heterogeneity in the upregulated group completely disappeared (I² = 0%), suggesting that the overall high heterogeneity was primarily attributable to differences in the direction of gene expression.

**Table 3:** Meta regression of OS

Covariates	t	*p*	Adj *R*-squared
Gene (UA)	−0.08	0.940	−7.89%
Gene express type (UA)	−6.30	<0.001	91.31%
Gene extraction method (UA)	0.94	0.363	2.56%
Data extraction method (UA)	−1.39	0.183	15.32%

UA = univariate analysis; Adj = adjusted

We also investigated the correlations between the glycolysis-related gene signature score and other clinical outcome parameters. The results demonstrated that high GGS correlated with worse DFS (HR = 2.02, 95% CI 1.54–2.64, *P* < 0.001; Fig. 2C) and RFS (HR = 2.38, 95% CI 1.39–4.08, *P* = 0.002; Fig. 2D). Subgroup analysis of DFS showed that upregulation of SLC2A2 (HR = 3.26, 95% CI 1.58–6.73, *P* = 0.001) and PKM (HR = 1.81, 95% CI 1.46–2.24, *P* < 0.001) was associated with poor prognosis in HCC. Subgroup analysis of RFS showed that upregulation of PKM (HR = 3.38, 95% CI 2.10–5.44, *P* < 0.001) was associated with worse prognosis in HCC. Sensitivity analyses indicated that the pooled HR for unadjusted OS (Figure S2A) and RFS (Figure S2B) were stable, as no single study substantially influenced the results. In contrast, the adjusted OS (Figure S2C) and DFS (Figure S2D) were sensitive to individual studies, particularly the study by Hu et al. [31]. After excluding this study, the pooled HRs were 1.98 (95% CI 1.66–2.37) for adjusted OS and 2.45 (95% CI 1.79–3.36) for DFS, highlighting potential instability. We formally assessed publication bias using the Egger test, which indicated significant bias for adjusted OS (*P* = 0.001), while unadjusted OS (*P* = 0.054), DFS (*P* = 0.125), and RFS (*P* = 0.310) showed no significant bias (Table S2). To further quantify the impact of publication bias, we performed a trim-and-fill analysis (Figure S2E), which imputed four missing studies. After adjustment, the pooled HR for adjusted OS increased markedly from 1.77 to 4.69 (95% CI 3.97–5.65), demonstrating that publication bias substantially inflated the original estimate.

Given the large number of studies involving *PKM*, we also conducted a sensitivity analysis focusing on *PKM*-related OS. Excluding Hu et al. [31] led to pooled HRs of 2.35 (95% CI 1.95–2.84) for unadjusted OS and 1.95 (95% CI 1.61–2.36) for adjusted OS. Publication bias was again detected (Figure S3, Table S3), and trim-and-fill analysis resulted in significant increases to 6.27 (95% CI 5.07–7.94) and 4.69 (95% CI 3.94–5.72), respectively, indicating that the adjusted OS results are particularly susceptible to publication bias.

### Clinicopathological correlates of glycolysis-related gene signature score in HCC

As shown in Figure S4 and Table S4, the glycolysis-related gene signature score was not significantly correlated with gender (OR = 0.89, 95% CI 0.72–1.09, *P* = 0.27), HBsAg (OR = 0.99, 95% CI 0.79–1.24, *P* = 0.96), cirrhosis (OR = 0.90, 95% CI 0.73–1.11, *P* = 0.34), tumor node (OR = 1.16, 95% CI 0.96–1.41, *P* = 0.13), TNM stage (OR = 1.65, 95% CI 0.95–2.86, *P* = 0.08), ALT (OR = 1.34, 95% CI 0.79–2.26, *P* = 0.27), lymph node metastasis (OR = 0.77, 95% CI 0.16–3.80, *P* = 0.75), hepatitis (OR = 0.74, 95% CI 0.44–1.25, *P* = 0.27), BCLC staging (OR = 1.39, 95% CI 0.90–2.15, *P* = 0.13), and tumor encapsulation (OR = 0.77, 95% CI 0.56–1.06, *P* = 0.11). However, a high glycolysis-related gene signature score was significantly correlated with several characteristics: age (OR = 0.85, 95% CI 0.73–0.99, *P* = 0.03, Figure S5A), tumor size (OR = 1.73, 95% CI 1.26–2.37, *P* < 0.001, Figure S5B), tumor differentiation (OR = 1.47, 95% CI 1.06–2.05, *P* = 0.02, Figure S5C), AFP (OR = 1.81, 95% CI 1.53–2.15, *P* < 0.001, Figure S5D), vascular invasion (OR = 2.37, 95% CI 1.95–2.88, *P* < 0.001, Figure S5E), clinical stage (OR = 3.67, 95% CI 1.25–10.76, *P* = 0.02, Figure S5F), and tumor embolus (OR = 3.49, 95% CI 1.98–6.15, *P* < 0.001, Figure S5G). Meanwhile, subgroup analysis showed that abnormal PKM expression correlated with age, tumor size, tumor differentiation, vascular invasion, tumor embolus, ALT, AFP, and clinical stage (Table S5).

A sensitivity analysis of the combined OR for the overall and GGS subgroups showed that the exclusion of any single study did not impact most of the results (Figure S6, S7, and S8). However, HBsAg (*P* = 0.011) and tumor size (*P* = 0.031) in the GGS subgroup showed a significant publication bias (Table S2). The trim-and-fill method was then applied (Figure S9), and the trimmed results were 2.27 (95% CI 2.21–3.48) and 4.47 (95% CI 3.97–5.65), respectively. The results show a significant reversal, indicating that the existing results may be unstable.

### Expression and prognostic value of GGS in the TCGA-LIHC cohort

**Fig. 3:**
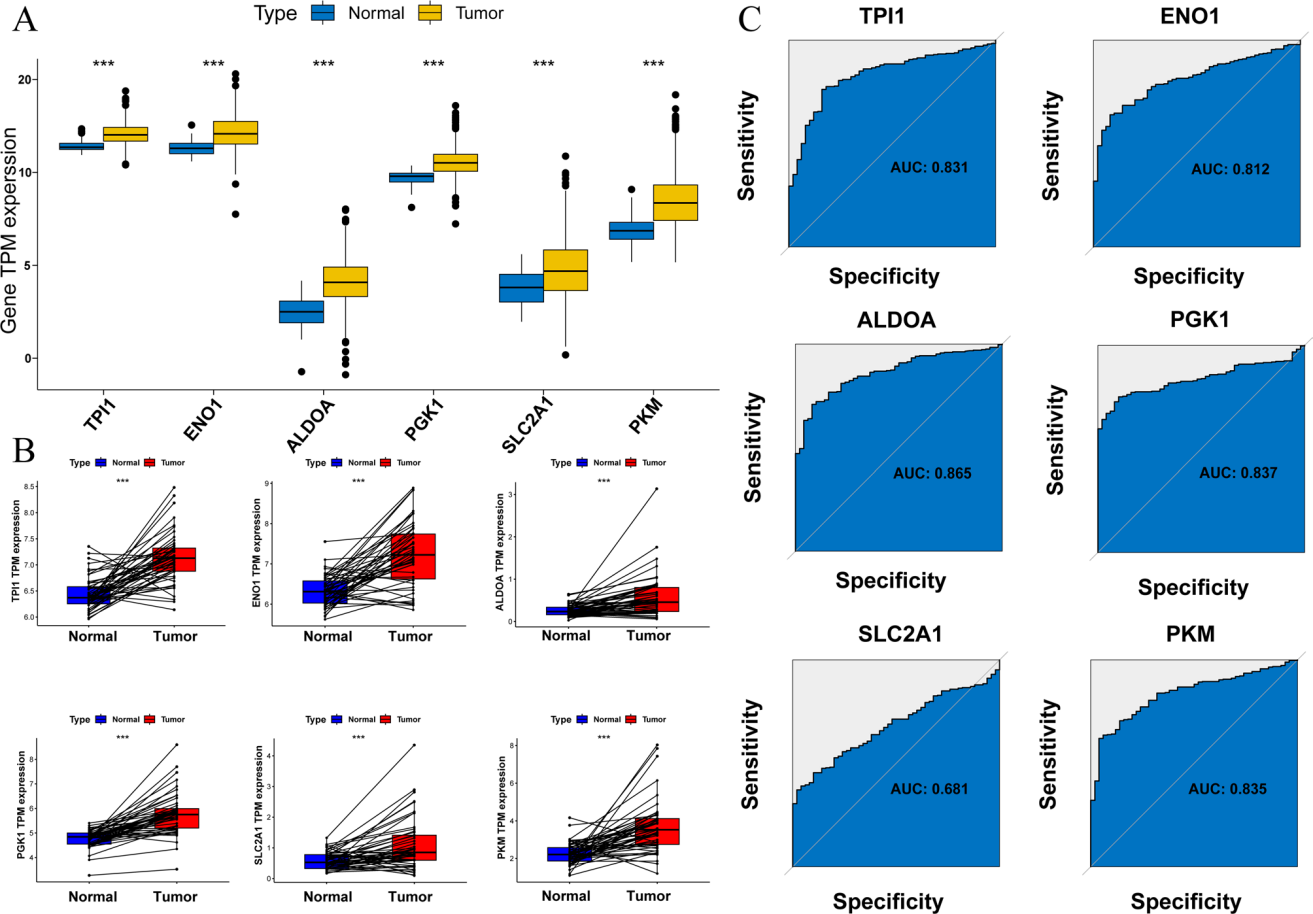
The tpm expression level of GGS in HCC. (**A**) normal and tumor tissues, (**B**) tumor and its paired tissues, (**C**) ROC graphics. Statistical significance is indicated as follows: *P* < 0.05 (*), *P* < 0.01 (**), and *P* < 0.001 (***)

First, we analyzed the glycolysis-related gene signature score in HCC tissues and found that GGS TPM expression levels were significantly higher in cancer tissues (*P* < 0.001) (Fig. 3A, B). We then evaluated the overall GGS values, which were all elevated, using ROC areas as follows: *TPI1* (AUC = 0.831), *ENO1* (AUC = 0.812), *ALDOA* (AUC = 0.865), *PGK1* (AUC = 0.837), *SLC2A1* (AUC = 0.681), and *PKM* (AUC = 0.835) (Fig. 3C).

**Fig. 4:**
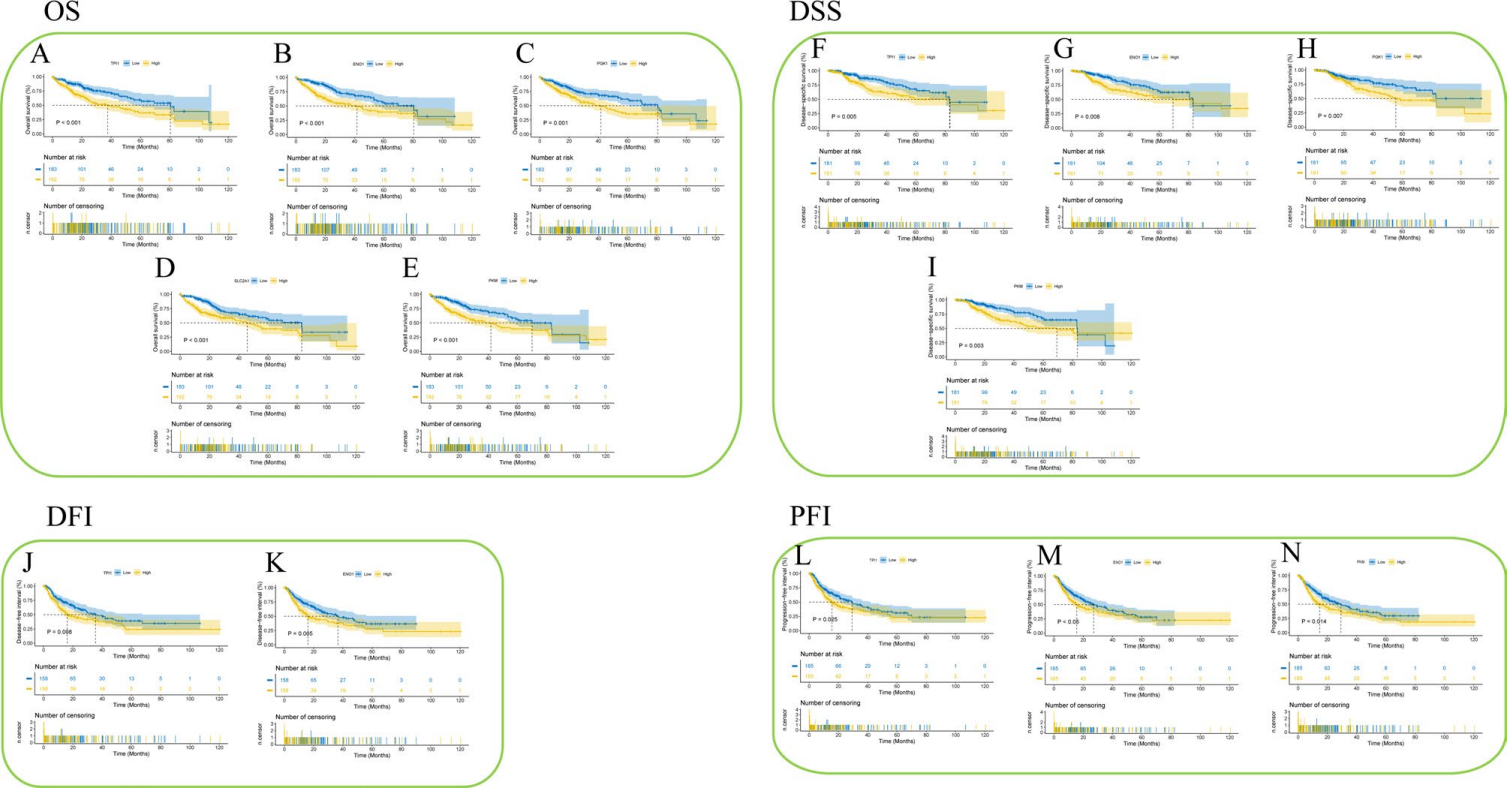
The Kaplan-Meier plotter method was used to draw the relationship between the high and low expression of GGS in HCC and the survival time (**A**) OS of TPI1, (**B**) OS of ENO1, (**C**) OS of PGK1, (**D**) OS of SLC2A1, (**E**) OS of PKM, (**F**) DSS of TPI1, (**G**) DSS of ENO1, (**H**) DSS of PGK1, (**I**) DSS of PKM, (**J**) DFI of TPI1, (**K**) DFI of ENO1, (**L**) PFI of TPI1, (**M**) PFI of ENO1, (**N**) PFI of PKM

The Kaplan-Meier method was used to further validate the correlation between the glycolysis-related gene signature score and clinical outcomes. As illustrated in Fig. 4, a high glycolysis-related gene signature score was associated with worse outcomes in patients with *TPI1* (OS, *P* < 0.001; DSS, *P* = 0.005; DFI, *P* = 0.008; PFI, *P* = 0.025), *ENO1* (OS, *P* < 0.001; DSS, *P* = 0.008; DFI, *P* = 0.005; PFI, *P* < 0.05), *PGK1* (OS: *P* = 0.001, DSS: *P* = 0.007), *SLC2A1* (OS: *P* < 0.001), and *PKM* (OS: *P* < 0.001, DSS: *P* = 0.003, PFI: *P* = 0.014).

### Glycolysis-related gene signature score with HCC clinicopathological characteristics and nomogram construction

**Fig. 5:**
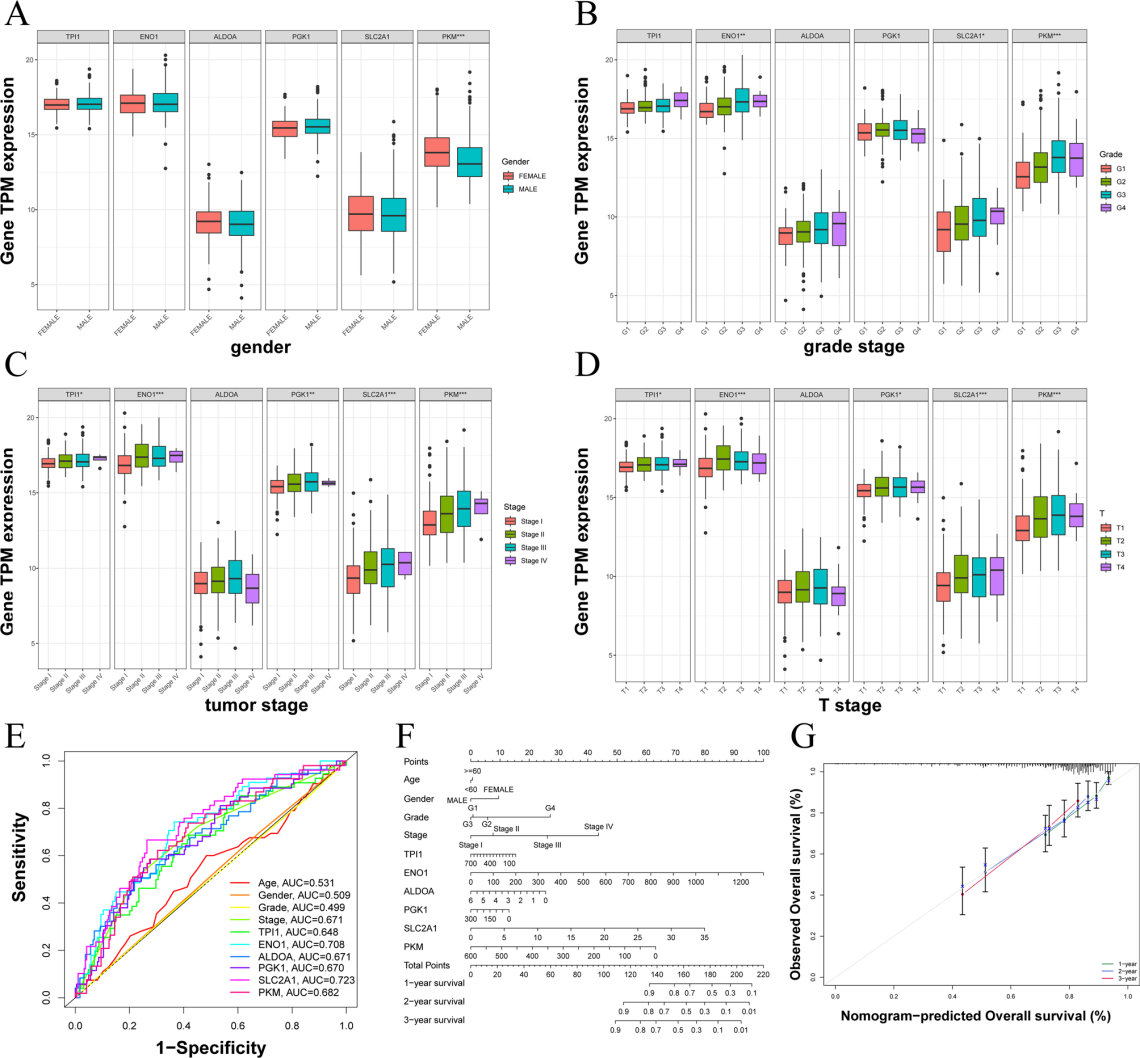
The relationship between the tpm expression of GGS in HCC and its clinical features. (**A**) gender, (**B**) grade stage, (**C**) tumor stage, (**D**) T stage, (**E**) ROC curves of clinical features and GGS, (**F**) nomogram, (**G**) calibration curves. Statistical significance is indicated as follows: *P* < 0.05 (*), *P* < 0.01 (**), and *P* < 0.001 (***)

We also investigated the association between GGS TPM expression and clinicopathological characteristics of HCC. Our analysis revealed differences in the glycolysis-related gene signature score across gender (Fig. 5A), grade stage (Fig. 5B), tumor stage (Fig. 5C), and T stage (Fig. 5D), while only *PKM* expression was found to be associated with age. Subsequently, ROC curves were generated for clinical features and GGS (Fig. 5E). The results indicated that most GGS markers (e.g., *ENO1*, AUC = 0.708; *SLC2A1*, AUC = 0.723; *PKM*, AUC = 0.682) exhibited higher discriminatory abilities than the traditional clinical features of HCC (age, sex, grade, and stage). Incorporating all GGS and clinical factors into the model, we constructed nomogram plots to predict the prognosis of patients with HCC at 1, 2, and 3 years (Fig. 5F). To evaluate the predictive accuracy of the nomogram, calibration curves were plotted for overall survival, comparing the predicted probabilities with the observed outcomes. As shown in Fig. 5G, the calibration curves closely aligned with the 45° reference line, indicating good agreement between predicted and actual survival probabilities across risk groups. These results suggest that the nomogram provides reliable prognostic predictions for HCC patients.

### In vitro validation results

**Fig. 6:**
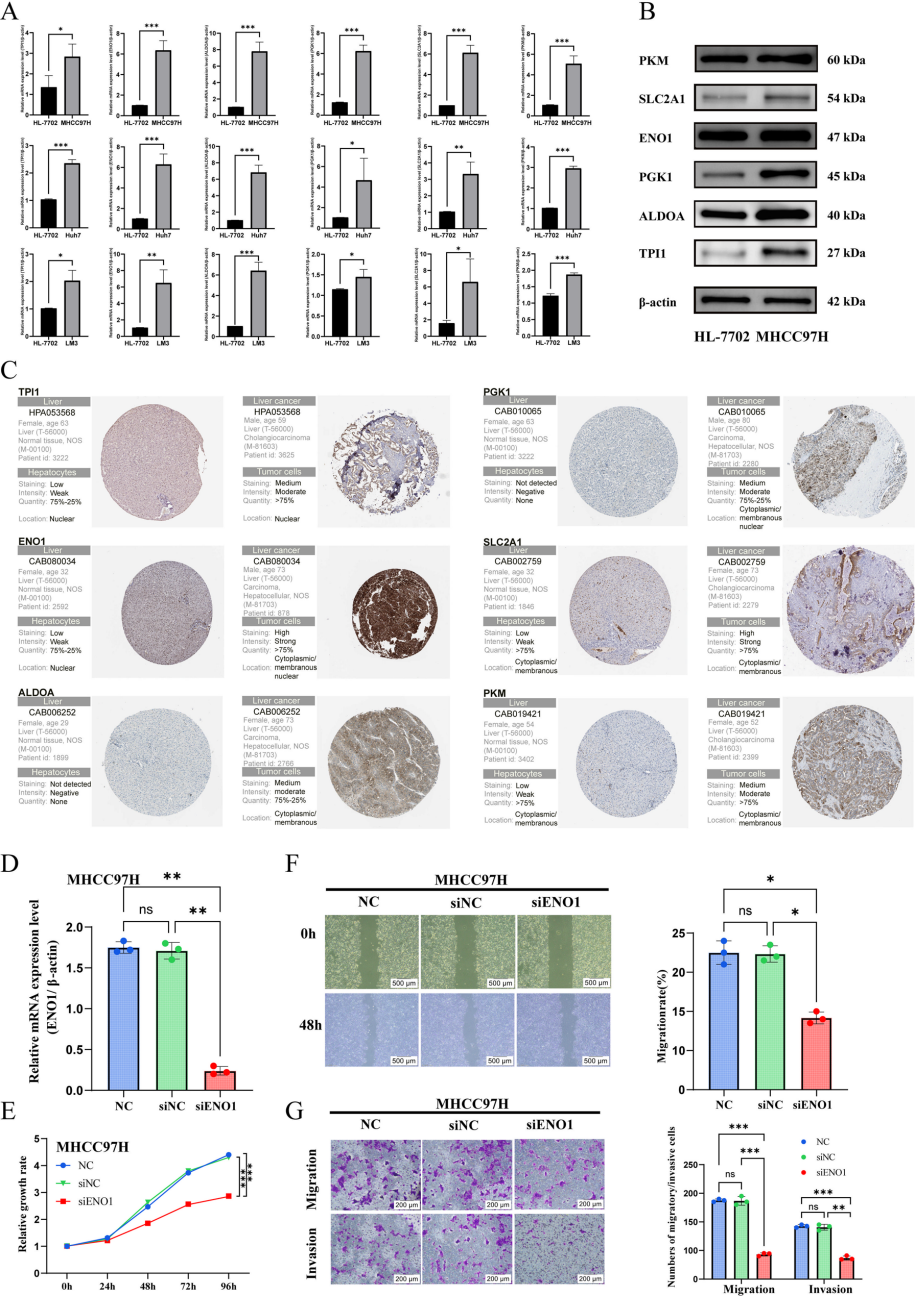
(**A**) RT-qPCR results of GGS using the HL-7702 cell line as the normal control group, (**B**) Western blot results of glycolysis-related gene signature score in normal and tumor cells, and (**C**) Immunohistochemistry analysis of glycolysis-related gene signature score in normal and tumor tissues. The mRNA levels following the knockdown of ENO1 expression in MHCC97H (**D**). (**E**) Assessment of the relationship between ENO1 knockout and cell proliferation using the MTT assay. Knockdown of ENO1 reduces the cell mobility of HCC in MHCC97H cells (**F**). Knockdown of ENO1 inhibits the migration and invasion of HCC in MHCC97H cells (**G**). Statistical significance is indicated as follows: *P* < 0.05 (*), *P* < 0.01 (**), and *P* < 0.001 (***)

RT-qPCR analyses in MHCC97H, Huh7, and HCC-LM3 cell lines showed elevated expression of most GGS compared with normal cell lines (Fig. 6A). Because the expression trend was most pronounced in MHCC97H cells, this line was selected for subsequent western blot analysis, which confirmed consistently higher GGS expression relative to normal cells (Fig. 6B). Immunohistochemistry data from the HPA database further demonstrated significant upregulation of most GGS in HCC tissues (Fig. 6C).

The successful knockdown of *ENO1* in MHCC97H cell lines was confirmed by qRT-PCR (Fig. 6D). MTT assays demonstrated that knocking down *ENO1* significantly inhibited the proliferation of HCC cells (Fig. 6E). Additionally, wound healing assays indicated that knocking down ENO1 decreased the migratory ability of HCC cells (Fig. 6F). Furthermore, transwell assays showed that knocking down ENO1 significantly reduced HCC cell migration and invasiveness (Fig. 6G).

### Functional enrichment and immune infiltration analysis of GGS

Subsequently, functional enrichment analysis was conducted using GGS. GO analysis revealed associations between GGS and glycolytic process, ATP synthesis, and carbon-oxygen lyase activity (Figure S10A). The KEGG results indicated that GGS might participate in various signaling pathways, including the HIF-1 signaling pathway and carbon metabolism in cancer (Figure S10B). Furthermore, the association between GGS and immune infiltration in HCC was assessed using ssGSEA. Immune cell types and functions differed in HCC (Figure S10C and D) and were closely linked to GGS (Figure S10E), suggesting potential avenues for further investigation. Moreover, immunoinfiltration analysis revealed a close correlation between GGS and common immune checkpoints in HCC (Figure S10F).

**Fig. 7:**
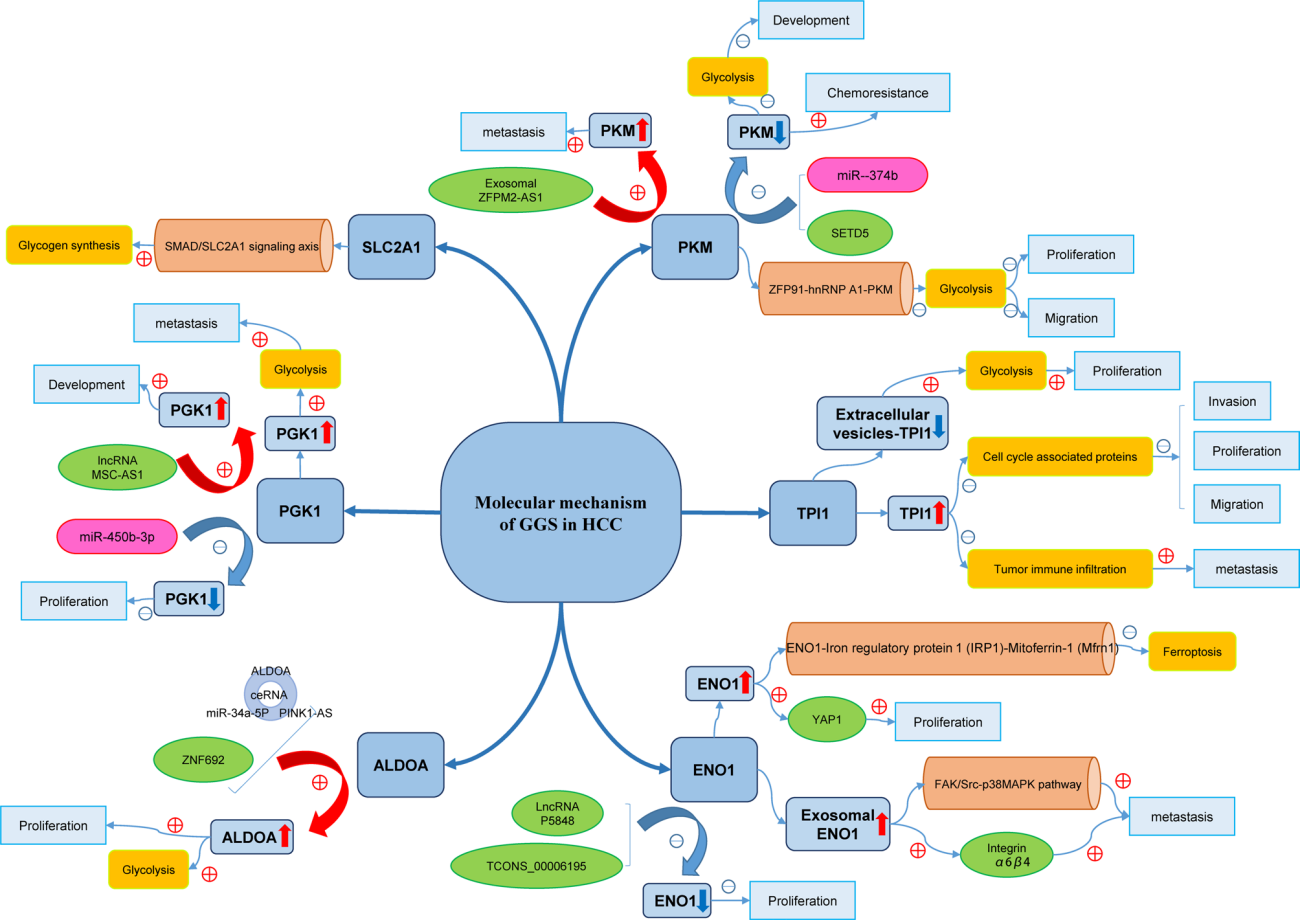
Hypothetical mechanistic model summarizing the regulatory role of GGS in HCC, based on literature evidence

Additionally, beyond these mechanisms, we synthesized the current understanding of the physiological and pathological roles of GGS in the regulation of HCC (Fig. 7, Table S6). These findings suggest that GGS may modulate the proliferation, invasion, and metastasis of HCC through the direct regulation of protein synthesis, interaction with non-coding RNAs, and regulation of glycolysis.

## Discussion

The relationship between glycolysis-related gene alterations and hepatocellular carcinoma (HCC) is not a simple unidirectional causal chain but rather a complex, mutually reinforcing vicious cycle. On one hand, genetic alterations can directly reprogram glycolytic metabolism, thereby initiating or accelerating hepatocarcinogenesis [38]. Several studies support this view. For example, Lu et al. reported that activation of the NF-κB signaling pathway enhances glycolysis in HCC [39], while Xia and colleagues demonstrated that N6-adenosine methyltransferase 5 (METTL5) stabilizes c-Myc protein by promoting *USP5* translation, thereby activating downstream glycolytic genes (e.g., *ENO1* and *PKM2*), reprogramming glucose metabolism, and promoting HCC proliferation and metastasis [40]. On the other hand, once HCC is established, tumor hypoxia persistently activates glycolysis-related pathways to alleviate oxygen deficiency and sustain rapid proliferation and metastasis [41]. Consistently, a 2021 study found that long non-coding RNA WAC-AS1 promotes glucose uptake and lactate production under hypoxic conditions by sponging miR-320d and regulating *ARPP19* expression [42]. Similarly, Fang et al. showed that activation of oncogenes such as *HRAS* further modulates glycolytic pathways to support tumor growth [43]. Thus, glycolysis-related gene dysregulation can act both as a cause and a consequence of HCC, with the two processes interacting throughout the tumor’s lifecycle to drive disease progression. Future research should aim to clarify this causal relationship through long-term prospective cohort studies in high-risk populations, such as patients with liver cirrhosis. Regular collection of biospecimens for multi-omics analyses may help capture dynamic changes in glycolysis-related genes during the transition from precancerous lesions to HCC, thereby elucidating their role in tumor initiation.

In our meta-analysis, the results demonstrated that a high glycolysis-related gene signature score was associated with worse OS, DFS, and RFS in HCC, which was largely consistent with the prognosis of GGS in bioinformatics analysis. Interestingly, our meta-analysis incorporating the study by Jiang et al. suggested that *TPI1* may act as a suppressor of HCC progression, which appears inconsistent with our bioinformatics findings indicating that *TPI1* upregulation is associated with poor prognosis. This discrepancy may be attributed to differences in study design and data sources. The study by Jiang et al. was based on a small cohort of 33 cases, which may not adequately capture the heterogeneity of HCC, whereas our bioinformatics analysis relied on large-scale patient datasets with greater statistical power. Moreover, accumulating evidence suggests that *TPI1* may exert context-dependent dual roles. While it can act as a suppressor under specific experimental conditions, deregulated *TPI1*—particularly in extracellular vehicles (EVs)—has been shown to promote aerobic glycolysis and enhance tumor aggressiveness [44, 45]. Since *TPI1* regulates the interconversion between *DHAP* and *G3P*, its dysregulation may disturb glucose metabolism and thereby facilitate cancer progression. Therefore, the divergence between our meta-analysis and bioinformatics results may reflect differences in the biological context, such as intracellular versus EV-associated *TPI1*, suggesting that *TPI1* may exert both tumor-suppressive and tumor-promoting functions depending on the microenvironment or molecular subtype of HCC. Future studies integrating large patient cohorts, distinct molecular subtypes, and in vivo validation are warranted to clarify the precise role of *TPI1* in HCC. Overall, our meta-analysis and bioinformatics analysis showed that GGS overexpression was positively correlated with tumor size, histological grade, and clinical stage. Biological assays have also shown that GGS is involved in tumor proliferation, invasion, and apoptosis [13, 22, 46], indicating that GGS can be used as a biological biomarker to assess the prognosis and clinical progression of HCC. In addition, our meta-analysis revealed that the results for unadjusted OS and RFS were relatively robust, whereas adjusted OS and DFS were more susceptible to the influence of individual studies, particularly the study by Hu et al. [31]. Significant publication bias was detected for adjusted OS, and after trim-and-fill correction, the pooled HR increased from 1.77 to 4.69, suggesting that the original estimate may have been substantially overestimated. Further analysis of *PKM* confirmed that adjusted OS was particularly vulnerable to bias. These findings indicate that caution should be exercised when interpreting adjusted OS and emphasize the importance of considering both unadjusted and adjusted results in prognostic evaluations.

Meanwhile, the DNA damage repair (DDR) pathway is a critical mechanism for maintaining genomic stability, and its dysfunction can contribute to the development of HCC [47]. Elucidating the relationship between GGS and DDR in HCC is therefore of particular importance, and several studies have provided supporting evidence. For instance, loss of the *ALDOA* gene has been shown to prolong the cell cycle and thereby suppress HCC cell proliferation, with cell-cycle arrest representing a key cellular response to DNA damage [48]. Chen et al. further demonstrated that *ALDOA* promotes pancreatic cancer proliferation by regulating DNA damage through the ATM–PLK1 pathway [49]. Li et al. reported that targeting *PGK1* with metabolic therapy activates compensatory DNA repair pathways, consequently reducing the sensitivity of tumor cells to chemotherapeutic agents [50]. Similarly, in oral squamous cell carcinoma, *TPI1* knockout enhanced cisplatin cytotoxicity [51]. Other studies have also indicated that *ENO1* and *SLC2A1* are associated with chemosensitivity in HCC [52, 53]. However, the precise mechanisms by which GGS genes influence DDR and chemoresistance in HCC remain unclear. Future investigations should include molecular biology experiments, such as overexpression or knockdown of *TPI1*, *PGK1*, and *PKM* in HCC cell lines, followed by assessment of DNA damage levels and the expression and activation of DNA repair proteins, to clarify the direct impact of glycolytic genes on DNA repair.

With the advancement of glycolysis research, several findings have already begun to show clinical relevance. First, glycolysis-related biomarkers can help predict the sensitivity of HCC to targeted therapies. For example, Pan et al. developed an aerobic glycolysis index (AGI) that reflects the level of aerobic glycolysis activity in HCC. Their study demonstrated that a high AGI is not only associated with poor differentiation and advanced stage but also accurately predicts tumor sensitivity to sorafenib, suggesting that AGI may serve as a biomarker to guide sorafenib treatment [54]. Second, glycolysis-related biomarkers may also predict the response to immunotherapy. Zhang et al. established an integrated prognostic signature combining immune and glycolytic pathways (IGRPS) and found that patients in the low-risk group exhibited better response rates to combined anti-CTLA4 and anti-PD-1 therapy [55]. It is important to note, however, that the causal relationship between glycolysis-related biomarkers and treatment response remains inconclusive. Most existing studies are based on correlation analyses, such as the association between high glycolytic activity and an immunosuppressive state. Nevertheless, such findings do not directly prove that targeting glycolysis can reverse immunosuppression or enhance immunotherapy efficacy. Further functional studies are required to elucidate the causal relationship and underlying mechanisms.

GGS has long been used in the treatment of HCC, and studies have found that inhibition of glycolysis-related gene signature score can increase the sensitivity of HCC cells to chemotherapeutic agents [56, 57] and improve the prognosis of patients [35]. To better understand the potential molecular mechanisms, we first analyzed the functional enrichment of GGS, and the results indicated the involvement of HIF-1 signaling pathway activation. An increasing number of studies have demonstrated that the HIF-1 signaling pathway is closely associated with various aspects of HCC. Activation of the HIF-1 signaling pathway leads to cell proliferation [58] and reduces chemoradiotherapy sensitivity of HCC [59–61], which could account for the poor clinical prognosis of HCC affected by glycolysis-related gene signature score. However, these mechanisms need to be further clarified by additional biological experiments. Additional studies have demonstrated that GGS promotes tumor progression by modulating HCC immunity [32], and the value of GGS in regulating the differentiation of cancer immune cells [62, 63] and as an immune target [64, 65] has also been reported. Our bioinformatics results showed that GGS is associated with imc, imf, and immune checkpoints in HCC, indicating that GGS could be a potential immune target for HCC.

Previous studies have shown that GGS is involved in various aspects of hepatocellular carcinoma (HCC). We summarized these as follows: First, GGS regulated the proliferation and migration of HCC by interacting with non-coding RNAs, including miRNA sponging and binding to lncRNAs [66–71]. Second, GGS regulates the progression of HCC through specific signaling pathways [21, 72, 73], such as the NF-κB and FAK/Src-p38MAPK pathways. In addition, GGS participates in specific metabolic processes in HCC, such as accelerating amino acid and protein synthesis [29, 74, 75], ferroptosis regulation [22], and apoptosis [76, 77]. Finally, GGS is related to the clinical treatment of HCC and may influence the efficacy of chemotherapeutic drugs [78]. Furthermore, the transcriptomic and proteomic results demonstrated the differential expression of GGS in HCC, which was consistent with our findings. Although mass spectrometry–based metabolomic analysis was not performed, we conducted functional assays focusing on [*ENO1*], a key glycolysis-related gene, which confirmed its role in regulating HCC cell proliferation and migration. These results provide additional support for the biological relevance of our findings, while future studies incorporating metabolomics will be needed for further validation. Although the nomogram demonstrated good predictive performance within the TCGA cohort, validation was limited to internal assessment, and external validation in independent cohorts was not performed. This represents a key limitation, and future studies are required to verify the model’s performance across diverse patient populations to ensure its generalizability and clinical applicability.

This study is the first to systematically evaluate the prognostic value of GGS in HCC by integrating meta-analysis, bioinformatics, and experimental validation. Nevertheless, several limitations should be acknowledged. First, inherent factors such as cancer subtype, detection methods, and follow-up duration may contribute to heterogeneity across included studies. Future investigations should adopt stricter inclusion and exclusion criteria and incorporate subgroup analyses to mitigate potential bias. Second, some studies did not directly report hazard ratios (HRs) and their 95% confidence intervals, requiring data extraction from Kaplan–Meier curves, which may compromise precision. Importantly, the lack of standardized cut-off values for high versus low glycolysis-related gene signature scores represents a critical limitation, as it reduces comparability across studies and constrains the clinical applicability of our findings. Establishing consistent, widely accepted thresholds in large, multicenter cohorts will be essential for validating and translating the prognostic significance of GGS. Finally, this study focused primarily on tumor tissue expression of GGS, and whether circulating GGS could serve as a non-invasive diagnostic or prognostic marker remains to be determined. In addition, the in vitro experiments in this study were limited to one HCC cell lines, which may not fully capture the heterogeneity of HCC. We therefore acknowledge this limitation, and further validation using a broader range of HCC cell lines as well as animal models will be necessary to enhance the robustness and translational potential of our findings.

## Conclusion

In conclusion, our study illustrates that GGS serves not only as a prognostic biomarker for HCC but is also closely associated with the clinical progression of the disease. Additionally, bioinformatic analyses suggest that GGS may exert its oncogenic effects through various mechanisms, including the regulation of HCC immunity and activation of relevant signaling pathways. While our analysis focused on six glycolysis-related genes and did not include other classical genes such as HK2, PFKM, and LDHA due to incomplete or inconsistent data, future studies incorporating a more comprehensive gene set and larger multi-omics datasets are warranted to validate and extend these findings. Overall, these results highlight glycolytic markers as promising therapeutic targets for patients with HCC and offer a novel perspective on the clinical application of glycolysis in this malignancy.

## Supplementary Information

Supplementary Material 1.

Supplementary Material 2.

## Data Availability

These data were derived from the following resources available in the public domain: [TCGA database; https://portal.gdc.cancer.gov/]. The original contributions presented in the study are included in the article/Supplementary Material. Further inquiries can be directed to the corresponding author.

## Abbreviations

HCC: Hepatocellular carcinoma
GGS: Glycolysis gene set
HR: Hazard ratio
OR: Odds ratio
CI: Confidence interval
OS: Overall survival
DFS: Disease-free survival
RFS: Relapse-free survival
ALDOA: Aldolase A
ENO1: Enolase 1
SLC2A1: Glucose transporter 1
PGK1: Phosphoglycerate kinase 1
TPI1: Triosephosphate isomerase 1
NOS: Newcastle-Ottawa scale
RT-qPCR: Quantitative reverse transcription polymerase chain reaction
IHC: Immunohistochemistry
UA: Univariate analysis
MA: Multivariate analysis
CP: Clinical parameters
Adj: Adjusted
NOS: Newcastle-Ottawa scale
GDSC: Genomics of drug sensitivity in cancer
CTRP: Cancer therapeutics response portal
GO: Gene ontology
KEGG: Kyoto encyclopedia of genes and genomes
imc: Immune cell composition
imf: Immune cell function
