# Supplementary material for: Prognostic evaluation of glycolysis markers in hepatocellular carcinoma: insights from meta-analysis and multi-omics approaches
Source: BMC Med Genomics. 2025 Nov 8;18:179. doi: 10.1186/s12920-025-02253-x (PMC12595624; doi:10.1186/s12920-025-02253-x)
Supplement: Supplementary file 2 — Supplementary Material 2. [file 12920_2025_2253_MOESM2_ESM.docx]

***SUPPLEMENTARY MATERIAL***

1. **Supplementary Figures**

**Figure S1:** Source analysis of heterogeneity for unadjusted OS: (A–D) meta-regression analyses; (E) subgroup analysis stratified by gene expression type.

**Figure S2:** Sensitivity analysis and publication bias for GGS: (A) unadjusted OS, (B) RFS, (C) adjusted OS, (D) DFS, (E) Trim-and-fill method for adjusted OS.

**Figure S3:** Sensitivity analysis, publication bias, and trim-and-fill method for PKM. (A) unadjusted OS, (B) adjusted OS.

**Figure S4:** Forest plot reflecting the relationship between GGS and clinicopathological characteristics of HCC patients. (A) gender, (B) HBsAg, (C) cirrhosis, (D) tumor node, (E) TNM stage, (F) ALT, (G) lymph node metastasis, (H) hepatitis, (I) BCLC stage, (J) tumor encapsulation.

**Figure S5:** Forest plot reflecting the relationship between GGS and clinicopathological characteristics of HCC patients. (A) Age, (B) tumor size (big vs. small), (C) tumor differentiation (poor vs. well), (D) AFP (high vs. low), (E) vascular invasion (yes vs. no), (F) clinical stage (III-IV vs. I-II), (G) tumor embolus (yes vs. no).

**Figure S6:** Sensitivity analysis for GGS with clinicopathological characteristics. (A) Age, (B) tumor size (big vs. small), (C) tumor differentiation (poor vs. well), (D) AFP (high vs. low), (E) vascular invasion (yes vs. no), (F) clinical stage (III-IV vs. I-II), (G) tumor embolus (yes vs. no).

**Figure S7:** Sensitivity analysis for GGS with clinicopathological characteristics. (A) gender, (B) HBsAg, (C) cirrhosis, (D) tumor node, (E) TNM stage, (F) ALT, (G) lymph node metastasis, (H) hepatitis, (I) BCLC stage, (J) tumor encapsulation.

**Figure S8:** Sensitivity analysis for PKM with clinicopathological characteristics. (A) age, (B) gender, (C) tumor size, (D) HBsAg, (E) tumor differentiation, (F) vascular invasion, (G) cirrhosis, (H) AFP, (I) hepatitis, (J) tumor node, (K) clinical stage, (L) tumor encapsulation, (M) TMN stage.

**Figure S9:** (A) Trim-and-fill method for OS in GGS and HBsAg, (B) Trim-and-fill method for OS in GGS and tumor size.

**Figure S10:** (A) GO analysis of GGS, (B) KEGG analysis of GGS, (C) imc in HCC, (D) imf in HCC, (E) The relationship between imc/imf and GGS, (F) the relationship between immune checkpoints and GGS. Statistical significance is indicated as follows: P < 0.05 (*), P < 0.01 (**), and P < 0.001 (***).

1. **Supplementary Table**

**Table S1.** Primer list for RT-qPCR.

**Table S2.** Publication bias of combined outcomes related to GSS.

**Table S3.** Publication bias of the combined PKM-related outcomes.

**Table S4.** Relationship between GSS expression and clinicopathological parameters.

**Table S5.** PKM upregulation in relation to clinicopathological parameters.

**Table S6**: Molecular mechanism of GGS in HCC.

**
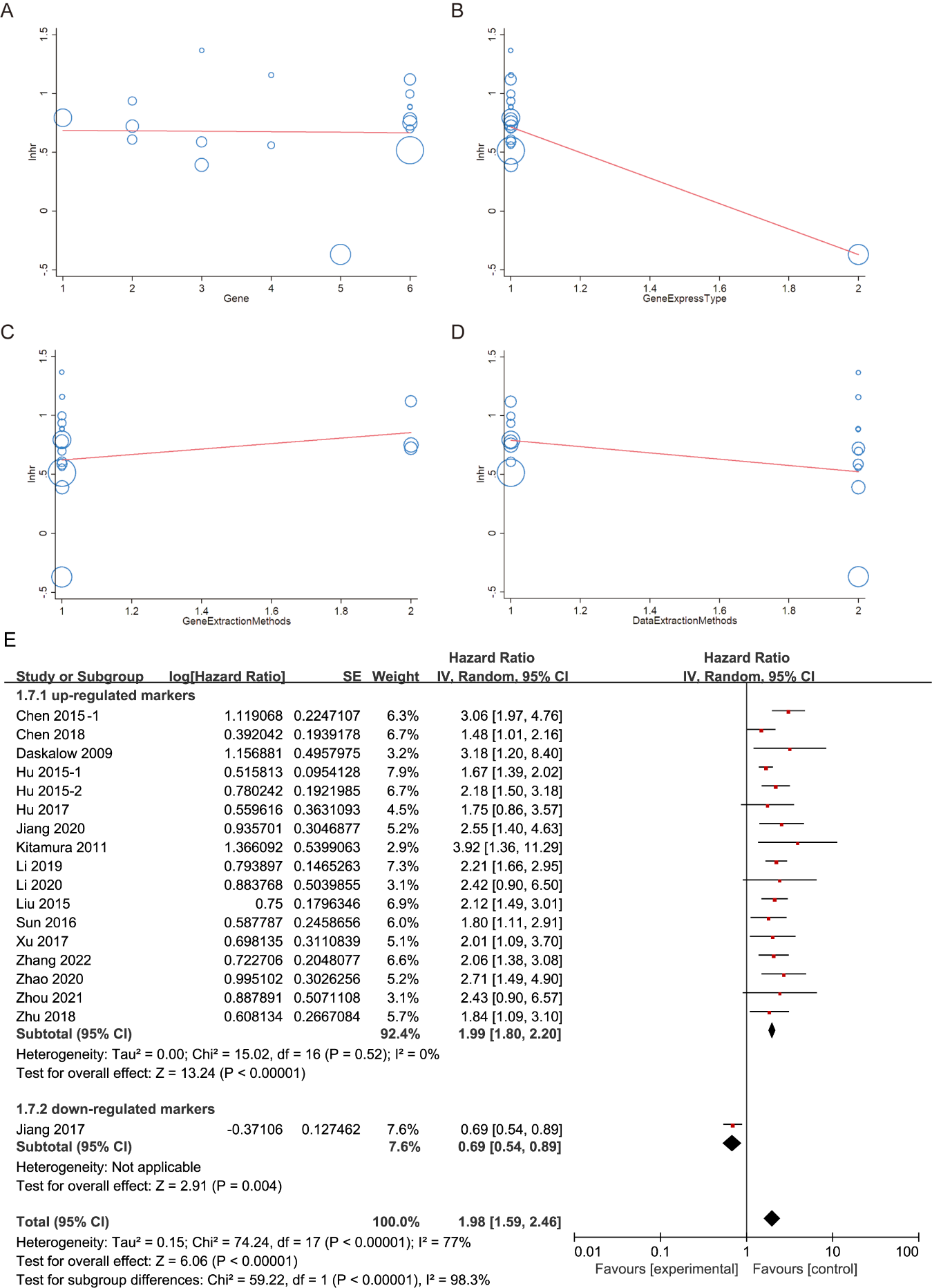
**

**Figure S1:** Source analysis of heterogeneity for unadjusted OS: (A–D) meta-regression analyses; (E) subgroup analysis stratified by gene expression type.

**
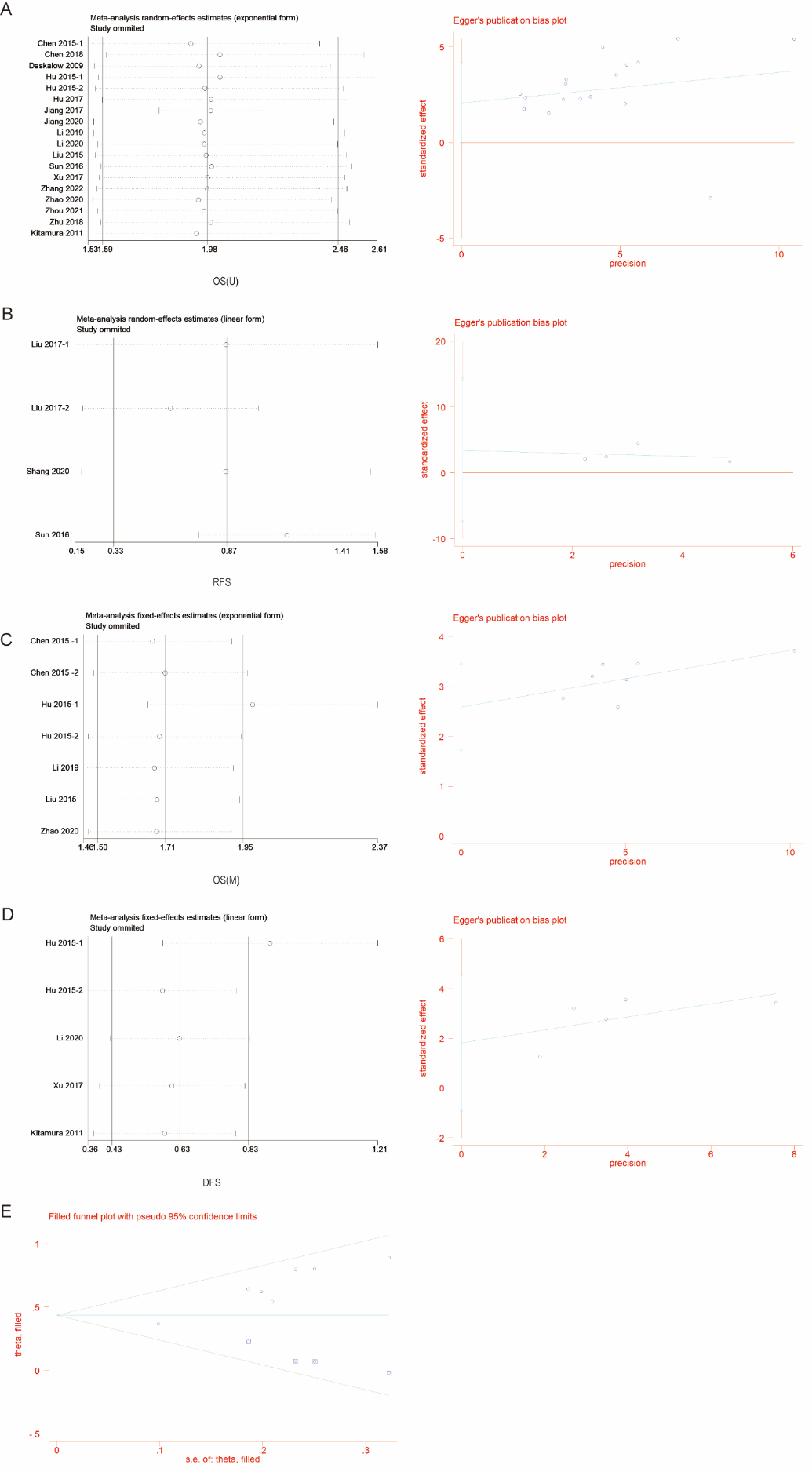
**

**Figure S2:** Sensitivity analysis and publication bias for GGS: (A) unadjusted OS, (B) RFS, (C) adjusted OS, (D) DFS, (E) Trim-and-fill method for adjusted OS.


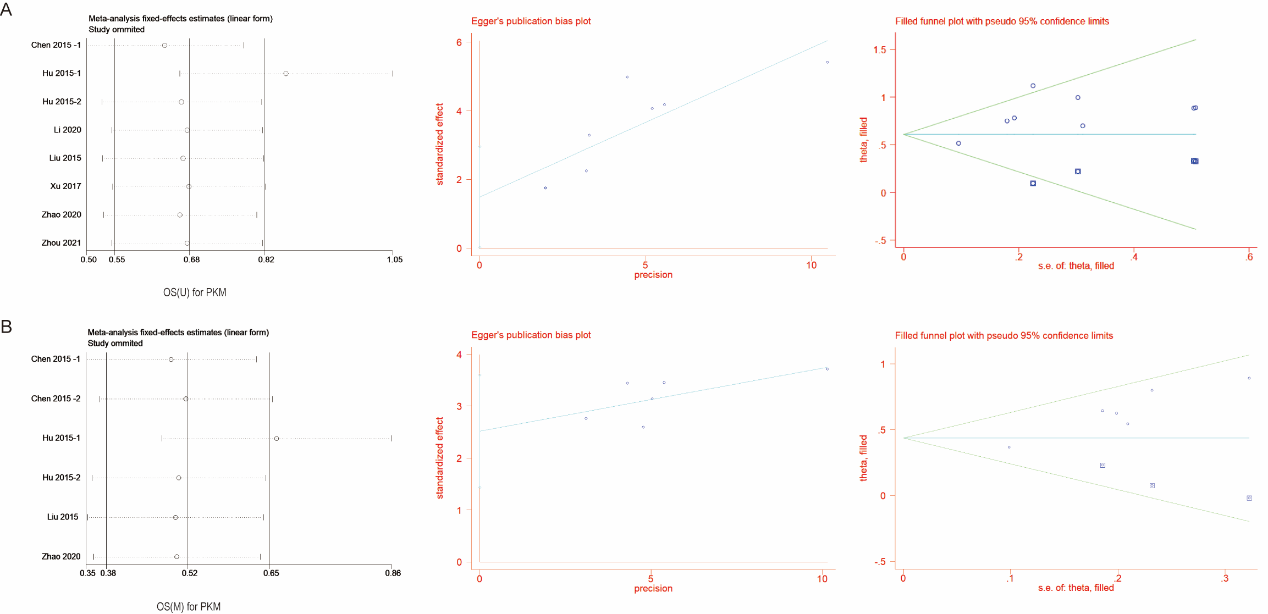


**Figure S3:** Sensitivity analysis, publication bias, and trim-and-fill method for PKM. (A) unadjusted OS, (B) adjusted OS.


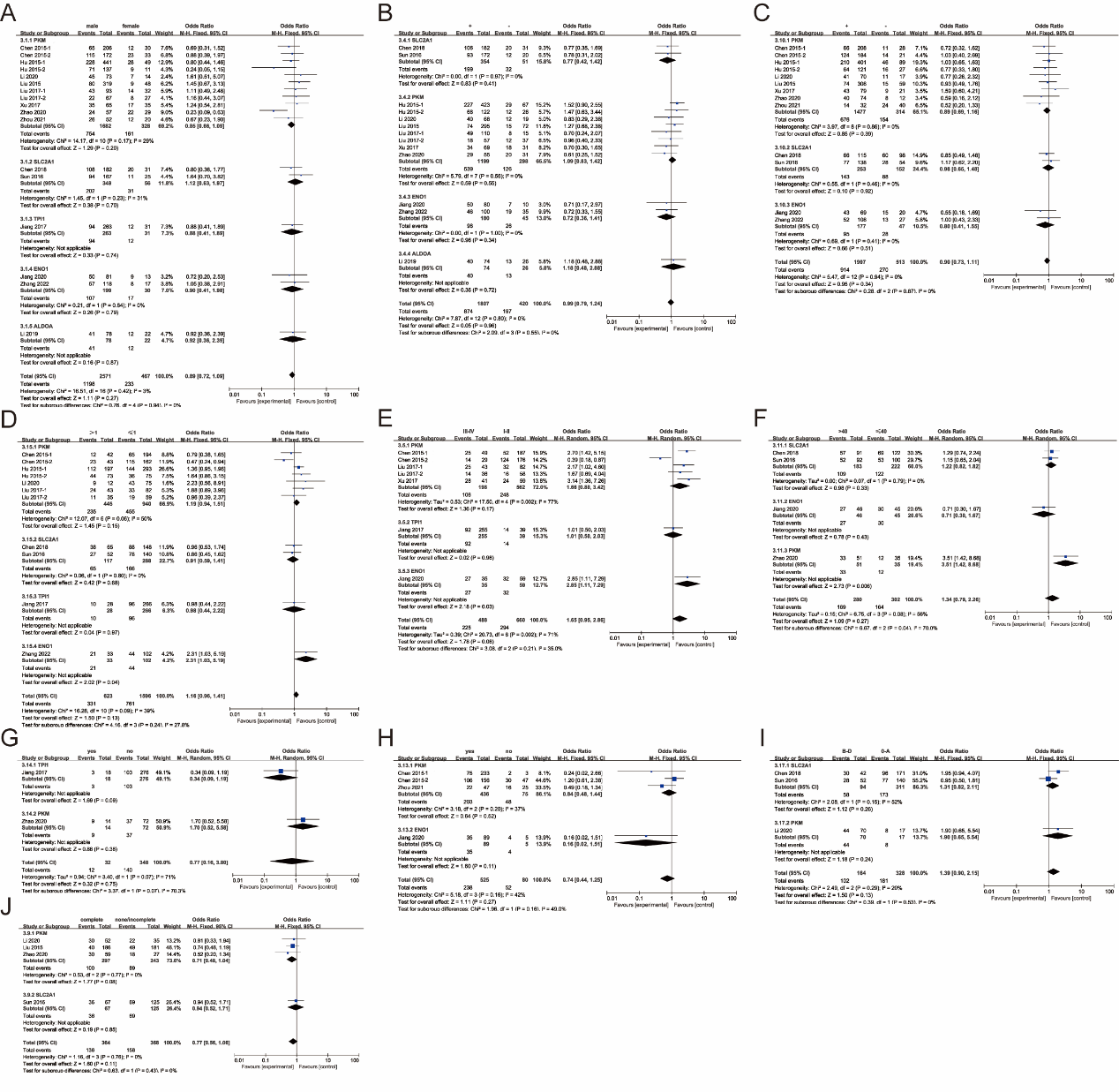


**Figure S4:** Forest plot reflecting the relationship between GGS and clinicopathological characteristics of HCC patients. (A) gender, (B) HBsAg, (C) cirrhosis, (D) tumor node, (E) TNM stage, (F) ALT, (G) lymph node metastasis, (H) hepatitis, (I) BCLC stage, (J) tumor encapsulation.


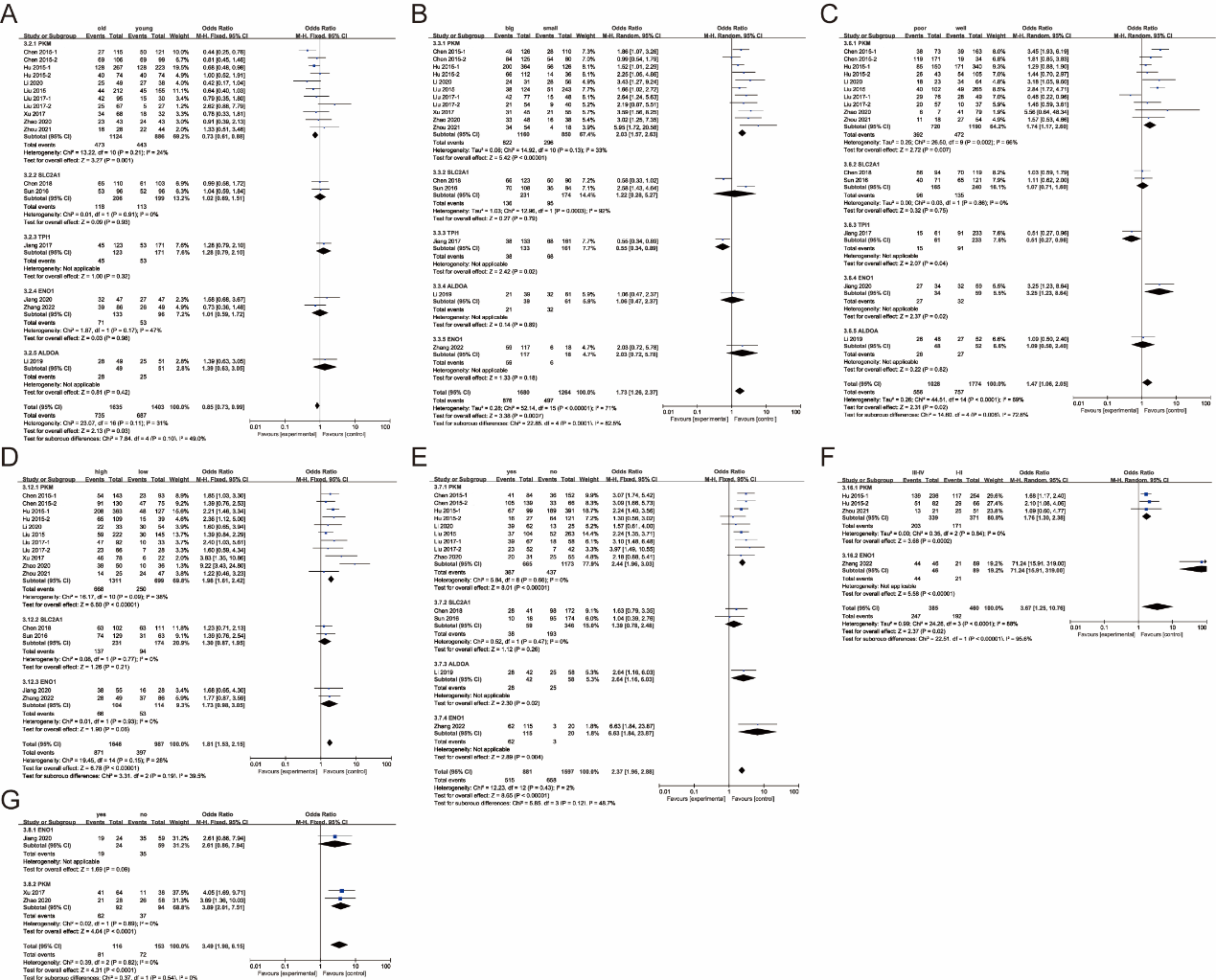


**Figure S5:** Forest plot reflecting the relationship between GGS and clinicopathological characteristics of HCC patients. (A) Age, (B) tumor size (big vs. small), (C) tumor differentiation (poor vs. well), (D) AFP (high vs. low), (E) vascular invasion (yes vs. no), (F) clinical stage (III-IV vs. I-II), (G) tumor embolus (yes vs. no).


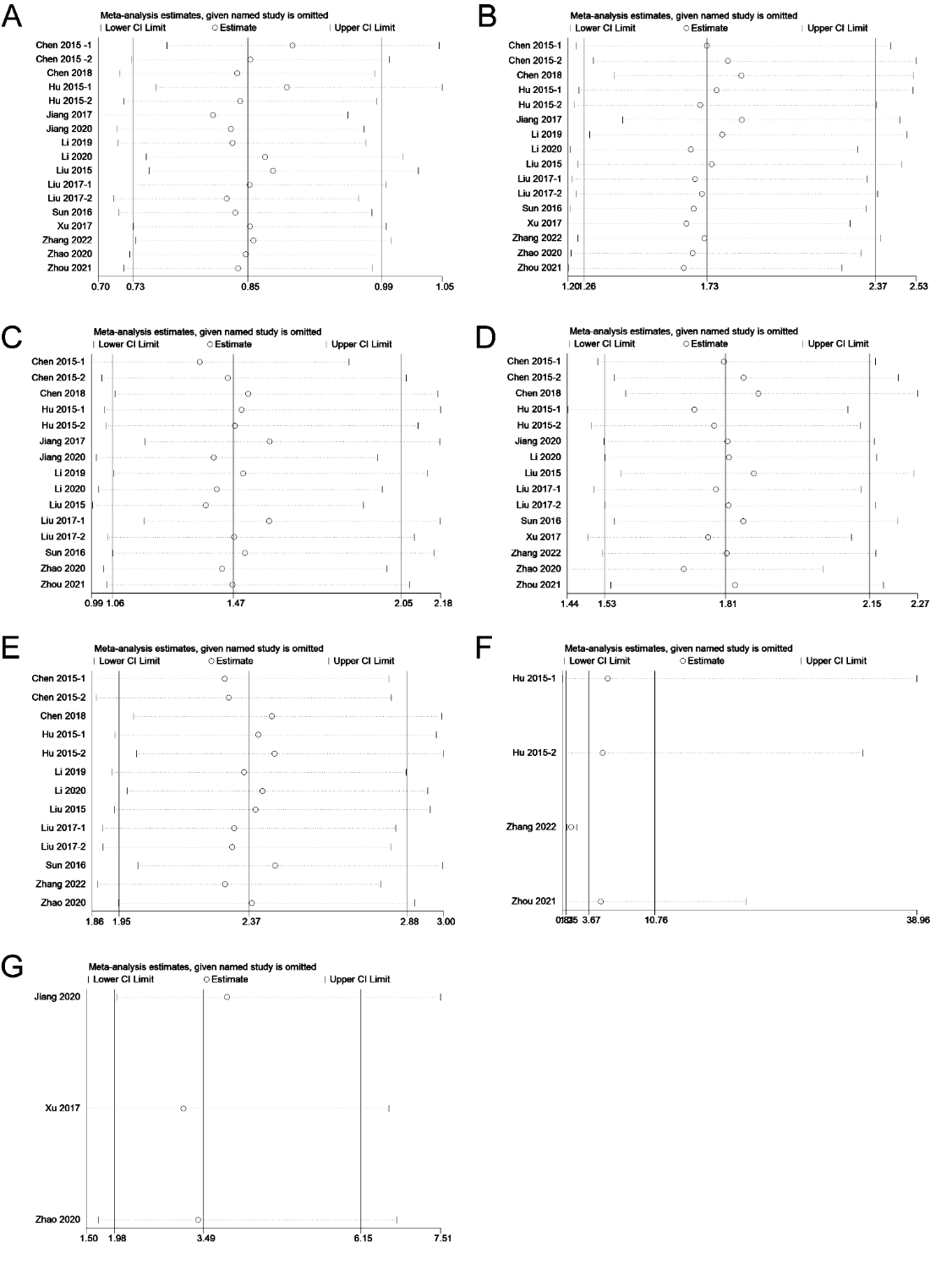


**Figure S6:** Sensitivity analysis for GGS with clinicopathological characteristics. (A) Age, (B) tumor size (big vs. small), (C) tumor differentiation (poor vs. well), (D) AFP (high vs. low), (E) vascular invasion (yes vs. no), (F) clinical stage (III-IV vs. I-II), (G) tumor embolus (yes vs. no).


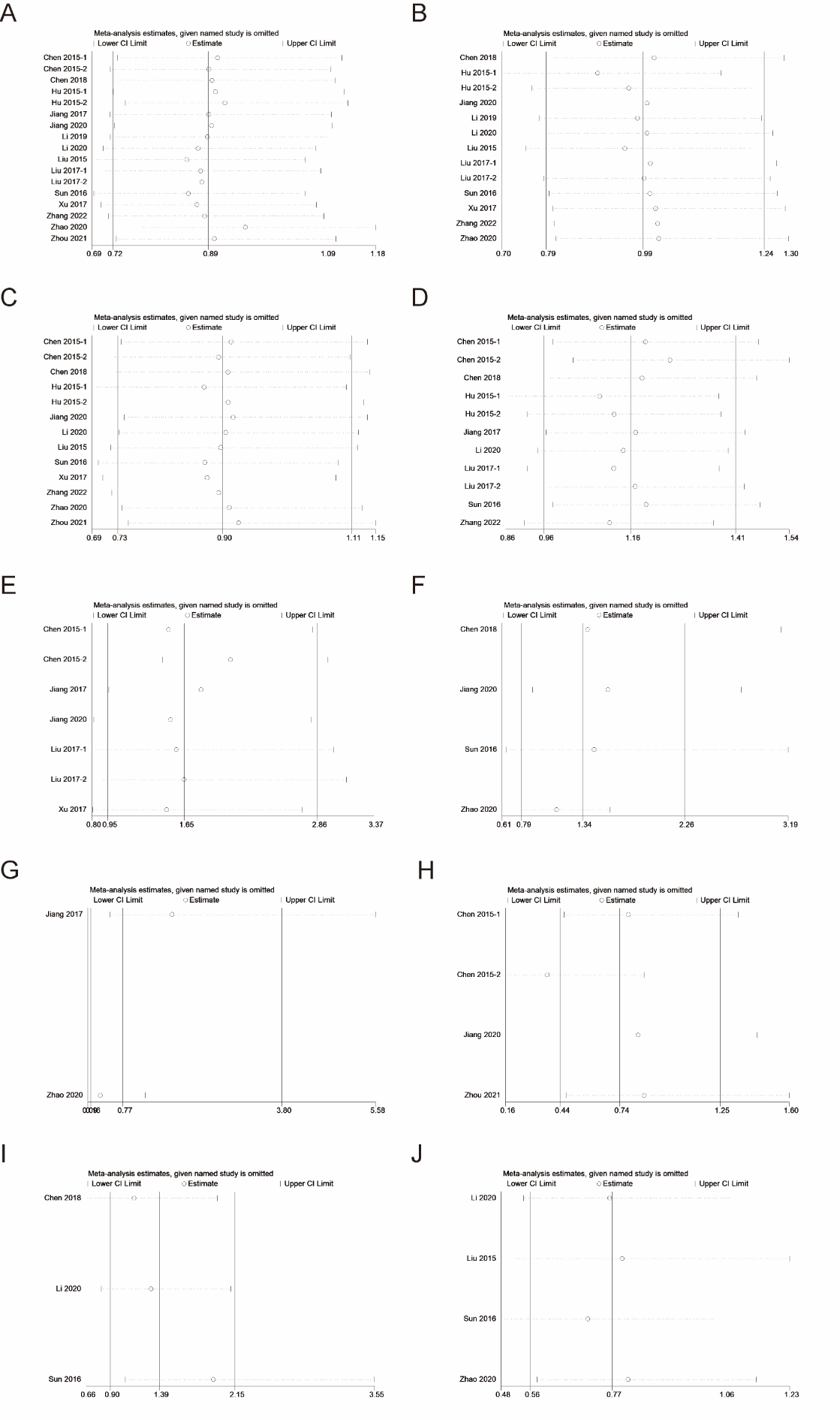


**Figure S7:** Sensitivity analysis for GGS with clinicopathological characteristics. (A) gender, (B) HBsAg, (C) cirrhosis, (D) tumor node, (E) TNM stage, (F) ALT, (G) lymph node metastasis, (H) hepatitis, (I) BCLC stage, (J) tumor encapsulation.


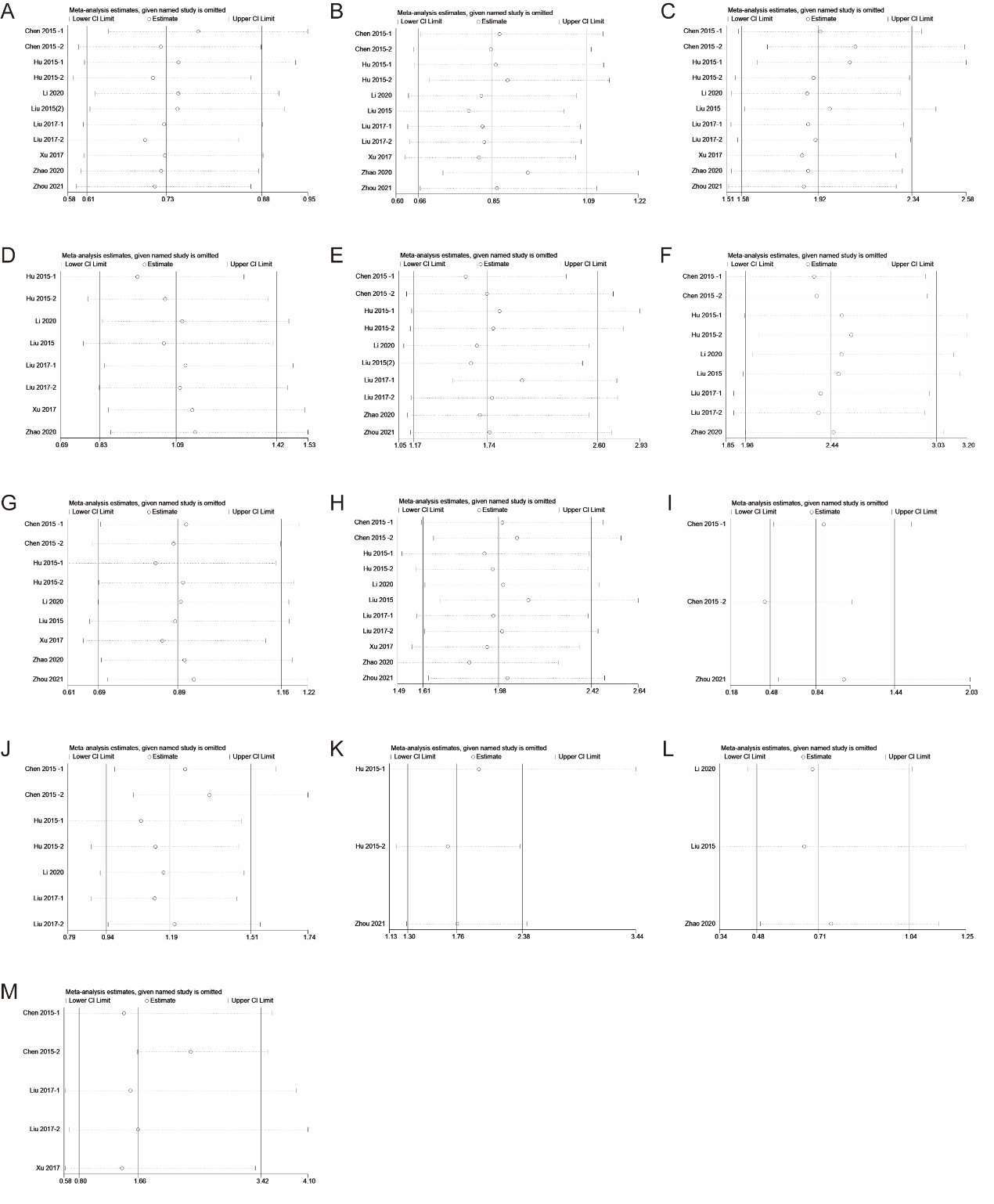


**Figure S8:** Sensitivity analysis for PKM with clinicopathological characteristics. (A) age, (B) gender, (C) tumor size, (D) HBsAg, (E) tumor differentiation, (F) vascular invasion, (G) cirrhosis, (H) AFP, (I) hepatitis, (J) tumor node, (K) clinical stage, (L) tumor encapsulation, (M) TMN stage.


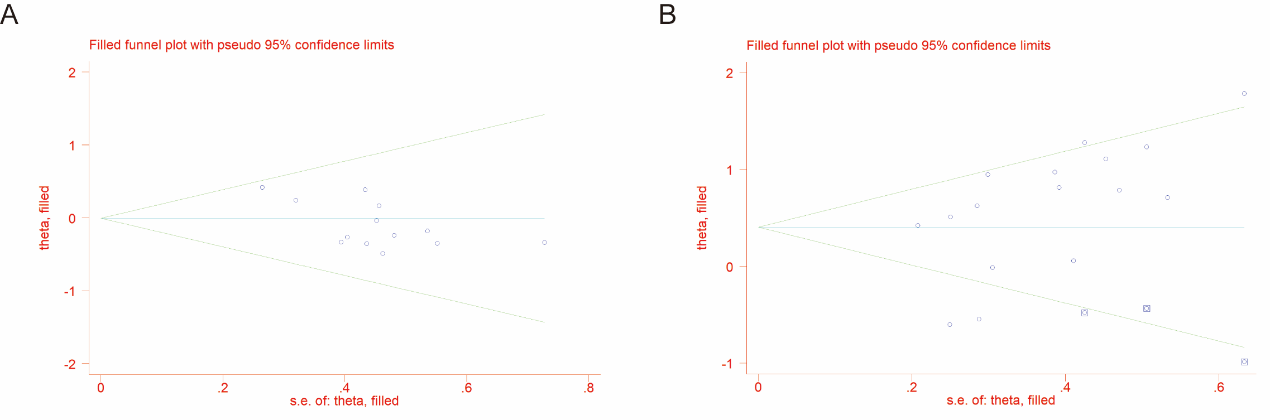


**Figure S9:** (A) Trim-and-fill method for OS in GGS and HBsAg, (B) Trim-and-fill method for OS in GGS and tumor size.


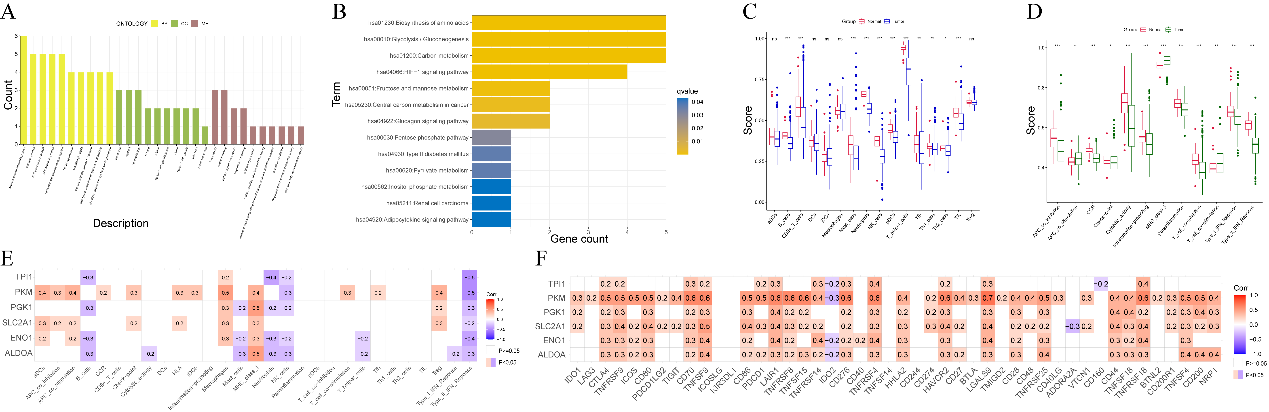


**Figure S10:** (A) GO analysis of GGS, (B) KEGG analysis of GGS, (C) imc in HCC, (D) imf in HCC, (E) The relationship between imc/imf and GGS, (F) the relationship between immune checkpoints and GGS. Statistical significance is indicated as follows: P < 0.05 (*), P < 0.01 (**), and P < 0.001 (***).

**Table S1.** Primer list for RT-qPCR.

| Gene Name | Forward primer | Reverse primer |
| --- | --- | --- |
| TPI1 | GCTGGCATCACTGAGAAGGTTG | CTCCATAAATGATACGGGTGCTCTG |
| ENO1 | GTTCAATGTCATCAATGGCGGTTC | TTTCCCAATAGCAGTCTTCAGCAG |
| ALDOA | ACCGAGAACACCGAGGAGAAC | GCTGGCAGATACTGGCATAACG |
| PGK1 | TGTGAAGATTACCTTGCCTGTTGAC | ACTTTATCCTCCGTGTTCCATTTGG |
| SLC2A1 | ATCGTCGTCGGCATCCTCATC | CGTGGAGTAATAGAAGACAGCGTTG |
| PKM | GTCATTCATCCGCAAGGCATCTG | GCACCGTCCAATCATCATCTTCTG |
| β-actin | GGACTTCGAGCAAGAGATGG | AGCACTGTGTTGGCGTACAG |

**Table S2.** Publication bias of combined outcomes related to GSS.

| Feature | Study | Egger test | |  | Harbord test | |
| --- | --- | --- | --- | --- | --- | --- |
|  |  | t | p |  | t | p |
| OS(UA) | 18 | 2.08 | 0.054 |  | - | - |
| OS(MA) | 7 | 7.73 | **0.001** |  | - | - |
| DFS | 5 | 2.12 | 0.125 |  | - | - |
| RFS | 4 | 1.35 | 0.310 |  | - | - |
| Age (old vs. young) | 17 | 1.56 | 0.139 |  | - | - |
| Gender (male vs. female) | 17 | -1.11 | 0.285 |  | - | - |
| Tumor size (big vs. small) | 16 | 2.40 | **0.031** |  | - | - |
| HBsAg (+ vs. -) | 13 | -3.05 | **0.011** |  | - | - |
| Tumor differentiation (poor vs. well) | 15 | 0.55 | 0.592 |  | - | - |
| Vascular invasion (yes vs. no) | 13 | -0.04 | 0.970 |  | - | - |
| Tumor embolus (yes vs. no) | 3 | - | - |  | -5.28 | 0.119 |
| Cirrhosis (+ vs. -) | 13 | -1.56 | 0.148 |  | - | - |
| ALT (>40 vs. ≤40) | 4 | - | - |  | 0.57 | 0.626 |
| AFP (high vs. low) | 15 | 1.23 | 0.241 |  | - | - |
| Hepatitis (yes vs. no) | 4 | - | - |  | 0.35 | 0.757 |
| Tumor node (>1 vs. ≤1) | 11 | -0.06 | 0.957 |  | - | - |
| Clinical stage (III-IV vs. I-II) | 4 | - | - |  | 0.09 | 0.940 |
| BCLC stage (B-D vs. 0-A) | 3 | - | - |  | -0.88 | 0.539 |
| Tumor encapsulation (complete vs. none/incomplete) | 4 | - | - |  | 0.27 | 0.830 |
| Lymph node metastasis (yes vs. no) | 2 | - | - |  | - | - |
| TNM stage (III-IV vs. I-II) | 7 | - | - |  | 0.45 | 0.673 |

**Table S3.** Publication bias of the combined PKM-related outcomes.

| Feature | No. of studies | Egger test | |  | Harbord test | |
| --- | --- | --- | --- | --- | --- | --- |
|  |  | t | p |  | t | p |
| OS(UA) | 8 | 2.48 | 0.048 |  | - | - |
| OS(MA) | 6 | 6.46 | 0.003 |  |  |  |
| Age (old vs. young) | 11 | -1.36 | 0.207 |  | - | - |
| Gender (male vs. female) | 11 | -1.11 | 0.296 |  | - | - |
| Tumor size (big vs. small) | 11 | -0.99 | 0.350 |  | - | - |
| HBsAg (+ vs. -) | 8 | - | - |  | -0.66 | 0.534 |
| Tumor differentiation (poor vs. well) | 10 | - | - |  | -1.03 | 0.333 |
| Vascular invasion (yes vs. no) | 9 | - | - |  | -1.84 | 0.109 |
| Tumor embolus (yes vs. no) | 2 | - | - |  | - | - |
| Cirrhosis (+ vs. -) | 9 | - | - |  | -2.13 | 0.071 |
| ALT (>40 vs. ≤40) | 1 | - | - |  | - | - |
| AFP (high vs. low) | 11 | 0.02 | 0.981 |  | - | - |
| Hepatitis (yes vs. no) | 3 | - | - |  | 0.34 | 0.790 |
| Tumor node (>1 vs. ≤1) | 7 | - | - |  | 0.20 | 0.853 |
| Clinical stage (III-IV vs. I-II) | 3 | - | - |  | 0.14 | 0.910 |
| BCLC stage (B-D vs. 0-A) | 1 | - | - |  | - | - |
| tumor encapsulation (complete vs. none/incomplete) | 3 | - | - |  | - | - |
| lymph node metastasis (yes vs. no) | 1 | - | - |  | - | - |
| TNM stage (III-IV vs. I-II) | 5 | - | - |  | 0.56 | 0.616 |

**Table S4.** Relationship between GSS expression and clinicopathological parameters.

| Feature | No. of studies | Test for association | | | Test for heterogeneity | | |
| --- | --- | --- | --- | --- | --- | --- | --- |
|  |  | OR | 95% CI | P | Chi2 | I^2^ | P |
| gender (male vs female) | 17 | 0.89 | 0.72-1.09 | 0.27 | 16.51 | 3% | 0.42 |
| age (old vs young) | 17 | 0.85 | 0.73-0.99 | 0.03 | 23.07 | 31% | 0.11 |
| tumor size (big vs small) | 16 | 1.73 | 1.26-2.37 | <0.001 | 52.14 | 71% | <0.001 |
| HBsAg (+ vs -) | 13 | 0.99 | 0.79-1.24 | 0.96 | 7.87 | 0% | 0.80 |
| TNM stage (III-IV vs I-II) | 7 | 1.65 | 0.95-2.86 | 0.08 | 20.73 | 71% | 0.002 |
| tumor differentiation (poor vs well) | 15 | 1.47 | 1.06-2.05 | 0.02 | 44.51 | 69% | <0.001 |
| vascular invasion (yes vs no) | 13 | 2.37 | 1.95-2.88 | <0.001 | 12.23 | 2% | 0.43 |
| tumor embolus (yes vs no) | 3 | 3.49 | 1.98-6.15 | <0.001 | 0.39 | 0% | 0.82 |
| tumor encapsulation (complete vs none/incomplete) | 4 | 0.77 | 0.56-1.06 | 0.11 | 1.16 | 0% | 0.76 |
| cirrhosis (+ vs -) | 13 | 0.90 | 0.73-1.11 | 0.34 | 5.47 | 0% | 0.94 |
| ALT (>40 vs ≤40) | 4 | 1.34 | 0.79-2.26 | 0.27 | 6.75 | 56% | 0.08 |
| AFP (high vs low) | 15 | 1.81 | 1.53-2.15 | <0.001 | 19.45 | 28% | 0.15 |
| hepatitis (yes vs no) | 4 | 0.74 | 0.44-1.25 | 0.27 | 5.18 | 42% | 0.27 |
| lymph node metastasis (yes vs no) | 2 | 0.77 | 0.16-3.80 | 0.75 | 0.94 | 71% | 0.07 |
| tumor node (>1 vs ≤1) | 11 | 1.16 | 0.96-1.41 | 0.13 | 16.28 | 39% | 0.09 |
| clinical stage (III-IV vs I-II) | 4 | 3.67 | 1.25-10.76 | 0.02 | 24.26 | 88% | <0.001 |
| BCLC stage (B-D vs 0-A) | 3 | 1.39 | 0.90-2.15 | 0.13 | 2.49 | 20% | 0.29 |

**Table S5.** PKM upregulation in relation to clinicopathological parameters.

| **Feature** | **No. of studies** | **Test for association** | | | **Test for heterogeneity** | | |
| --- | --- | --- | --- | --- | --- | --- | --- |
|  |  | **OR** | **95% CI** | **p** | **Chi2** | **I^2^** | **p** |
| Gender (male vs. female) | 11 | 0.85 | 0.66-1.09 | 0.20 | 14.17 | 29% | 0.17 |
| Age (old vs. young) | 11 | 0.73 | 0.61-0.88 | **0.001** | 13.22 | 24% | 0.21 |
| Tumor size (big vs. small) | 11 | 2.03 | 1.57-2.63 | **<0.001** | 14.92 | 33% | 0.13 |
| HBsAg (+ vs. -) | 8 | 1.09 | 0.83-1.42 | 0.55 | 5.79 | 0% | 0.56 |
| TNM stage (III-IV vs. I-II) | 5 | 1.66 | 0.80-3.42 | 0.17 | 17.50 | 77% | 0.002 |
| Tumor differentiation (poor vs. well) | 10 | 1.74 | 1.17-2.60 | **0.007** | 26.50 | 66% | 0.002 |
| Vascular invasion (yes vs. no) | 9 | 2.44 | 1.96-3.04 | **<0.001** | 5.84 | 0% | 0.66 |
| Tumor embolus (yes vs. no) | 2 | 3.89 | 2.02-7.51 | **<0.001** | 0.02 | 0% | 0.89 |
| Tumor encapsulation (complete vs. none/incomplete) | 3 | 0.71 | 0.48-1.04 | 0.08 | 0.53 | 0% | 0.77 |
| Cirrhosis (+ vs. -) | 9 | 0.89 | 0.69-1.16 | 0.39 | 3.97 | 0% | 0.86 |
| ALT (>40 vs. ≤40) | 1 | 3.51 | 1.42-8.68 | **0.006** | - | - | - |
| AFP (high vs. low) | 11 | 1.98 | 1.61-2.42 | **<0.001** | 16.17 | 38% | 0.09 |
| Hepatitis (yes vs. no) | 3 | 0.84 | 0.48-1.44 | 0.52 | 3.18 | 37% | 0.20 |
| Lymph node metastasis (yes vs. no) | 1 | 1.70 | 0.52-5.58 | 0.38 | - | - | - |
| Tumor node (>1 vs. ≤1) | 7 | 1.19 | 0.94-1.51 | 0.15 | 12.07 | 50% | 0.06 |
| Clinical stage (III-IV vs. I-II) | 3 | 1.76 | 1.30-2.38 | **<0.001** | 0.35 | 0% | 0.84 |
| BCLC stage (B-D vs. 0-A) | 1 | 1.90 | 0.65-5.54 | 0.24 | - | - | - |

**Table S6**: Molecular mechanism of GGS in HCC.

| Gene Symbol | Regulatory relationship | Phenotype | Reference |
| --- | --- | --- | --- |
| TPI1 | Cell cycle associated protein | Proliferation, migration, and invasion | [1] |
|  | Glycolysis | Proliferation | [2] |
|  | Tumor immune infiltration | Metastasis | [3] |
| ENO1 | ENO1-Iron regulatory protein 1 (IRP1)-Mitoferrin-1 (Mfrn1) | Ferroptosis | [4] |
|  | YAP1 | Proliferation | [5] |
|  | FAK/Src-p38MAPK pathway,Integrin α6β4 | Metastasis | [6] |
|  | LncRNA TCONS_00006195 | Proliferation | [7] |
|  | LncRNA P5848 | Proliferation | [8] |
| ALDOA | miR-34a-5P, PINK1-AS | Proliferation | [9] |
|  | ZNF692, Glycolysis | Progression | [10] |
|  | mRNA translation and protein biosynthesis | Progression | [11] |
| PGK1 | Glycolysis | Metastasis | [12] |
|  | LncRNA MSC-AS1 | Development | [13] |
|  | MiR-450b-3p | Proliferation | [14] |
| SLC2A1 | SMAD/SLC2A1 signaling axis | Glycogen synthesis | [15] |
| PKM | ZFP91-hnRNP A1-PKM, Glycolysis | Proliferation, migration | [16] |
|  | ZFPM2-AS1 | Metastasis | [17] |
|  | miR-374b | Chemoresistance | [18] |
|  | SETD5, Glycolysis | Development | [19] |

1. Jiang H, Ma N, Shang Y, Zhou W, Chen T, Guan D, Li J, Wang J, Zhang E, Feng Y, Yin F, Yuan Y, Fang Y, Qiu L, Xie D and Wei D (2017) Triosephosphate isomerase 1 suppresses growth, migration and invasion of hepatocellular carcinoma cells. Biochem Biophys Res Commun 482:1048-1053. doi: 10.1016/j.bbrc.2016.11.156

2. Liu BHM, Tey SK, Mao X, Ma APY, Yeung CLS, Wong SWK, Ng TH, Xu Y, Yao Y, Fung EYM, Tan KV, Khong PL, Ho DW, Ng IO, Tang AHN, Cai SH, Yun JP and Yam JWP (2021) TPI1-reduced extracellular vesicles mediated by Rab20 downregulation promotes aerobic glycolysis to drive hepatocarcinogenesis. J Extracell Vesicles 10:e12135. doi: 10.1002/jev2.12135

3. Liang Y, Zhong D, Yang Q, Tang Y, Qin Y, Su Y, Huang X and Shang J (2024) Single-Cell RNA Sequencing Revealed That the Enrichment of TPI1(+) Malignant Hepatocytes Was Linked to HCC Metastasis and Immunosuppressive Microenvironment. J Hepatocell Carcinoma 11:373-383. doi: 10.2147/jhc.S453249

4. Zhang T, Sun L, Hao Y, Suo C, Shen S, Wei H, Ma W, Zhang P, Wang T, Gu X, Li ST, Chen Z, Yan R, Zhang Y, Cai Y, Zhou R, Jia W, Huang F, Gao P and Zhang H (2022) ENO1 suppresses cancer cell ferroptosis by degrading the mRNA of iron regulatory protein 1. Nat Cancer 3:75-89. doi: 10.1038/s43018-021-00299-1

5. Sun L, Suo C, Zhang T, Shen S, Gu X, Qiu S, Zhang P, Wei H, Ma W, Yan R, Chen R, Jia W, Cao J, Zhang H and Gao P (2023) ENO1 promotes liver carcinogenesis through YAP1-dependent arachidonic acid metabolism. Nat Chem Biol 19:1492-1503. doi: 10.1038/s41589-023-01391-6

6. Jiang K, Dong C, Yin Z, Li R, Mao J, Wang C, Zhang J, Gao Z, Liang R, Wang Q and Wang L (2020) Exosome-derived ENO1 regulates integrin α6β4 expression and promotes hepatocellular carcinoma growth and metastasis. Cell Death Dis 11:972. doi: 10.1038/s41419-020-03179-1

7. Yu S, Li N, Huang Z, Chen R, Yi P, Kang R, Tang D, Hu X and Fan X (2018) A novel lncRNA, TCONS_00006195, represses hepatocellular carcinoma progression by inhibiting enzymatic activity of ENO1. Cell Death Dis 9:1184. doi: 10.1038/s41419-018-1231-4

8. (2020) Expression of Concern: Targetting an LncRNA P5848-ENO1 axis inhibits tumor growth in hepatocellular carcinoma. Biosci Rep 40. doi: 10.1042/bsr-20180896_eoc

9. Wang J, Zhang HM, Dai ZT, Huang Y, Liu H, Chen Z, Wu Y and Liao XH (2022) MKL-1-induced PINK1-AS overexpression contributes to the malignant progression of hepatocellular carcinoma via ALDOA-mediated glycolysis. Sci Rep 12:21283. doi: 10.1038/s41598-022-24023-w

10. Meng W, Lu X, Wang G, Xiao Q and Gao J (2024) ZNF692 drives malignant development of hepatocellular carcinoma cells by promoting ALDOA-dependent glycolysis. Funct Integr Genomics 24:53. doi: 10.1007/s10142-024-01326-x

11. Song J, Li H, Liu Y, Li X, Shi Q, Lei QY, Hu W, Huang S, Chen Z and He X (2023) Aldolase A Accelerates Cancer Progression by Modulating mRNA Translation and Protein Biosynthesis via Noncanonical Mechanisms. Adv Sci (Weinh) 10:e2302425. doi: 10.1002/advs.202302425

12. Xie H, Tong G, Zhang Y, Liang S, Tang K and Yang Q (2017) PGK1 Drives Hepatocellular Carcinoma Metastasis by Enhancing Metabolic Process. Int J Mol Sci 18. doi: 10.3390/ijms18081630

13. Cao C, Zhong Q, Lu L, Huang B, Li J, Meng L and Wei H (2020) Long noncoding RNA MSC-AS1 promotes hepatocellular carcinoma oncogenesis via inducing the expression of phosphoglycerate kinase 1. Cancer Med 9:5174-5184. doi: 10.1002/cam4.3080

14. Chen Z, Zhuang W, Wang Z, Xiao W, Don W, Li X and Chen X (2019) MicroRNA-450b-3p inhibits cell growth by targeting phosphoglycerate kinase 1 in hepatocellular carcinoma. J Cell Biochem 120:18805-18815. doi: 10.1002/jcb.29196

15. Zhong J, Tian L, Gou Y, Zhao P, Dong X, Guo M, Zhao G, Li A, Hao A, He TC and Fan J (2023) BMP4 upregulates glycogen synthesis through the SMAD/SLC2A1 (GLUT1) signaling axis in hepatocellular carcinoma (HCC) cells. Cancer Metab 11:9. doi: 10.1186/s40170-023-00310-6

16. Chen D, Wang Y, Lu R, Jiang X, Chen X, Meng N, Chen M, Xie S and Yan GR (2020) E3 ligase ZFP91 inhibits Hepatocellular Carcinoma Metabolism Reprogramming by regulating PKM splicing. Theranostics 10:8558-8572. doi: 10.7150/thno.44873

17. Ji W, Bai J and Ke Y (2023) Exosomal ZFPM2-AS1 contributes to tumorigenesis, metastasis, stemness, macrophage polarization, and infiltration in hepatocellular carcinoma through PKM mediated glycolysis. Environ Toxicol 38:1332-1346. doi: 10.1002/tox.23767

18. Zhang M, Zhang H, Hong H and Zhang Z (2019) MiR-374b re-sensitizes hepatocellular carcinoma cells to sorafenib therapy by antagonizing PKM2-mediated glycolysis pathway. Am J Cancer Res 9:765-778.

19. Park M, Moon B, Kim JH, Park SJ, Kim SK, Park K, Kim J, Kim SY, Kim JH and Kim JA (2022) Downregulation of SETD5 Suppresses the Tumorigenicity of Hepatocellular Carcinoma Cells. Mol Cells 45:550-563. doi: 10.14348/molcells.2022.0009

1. **Experimental raw data**

**
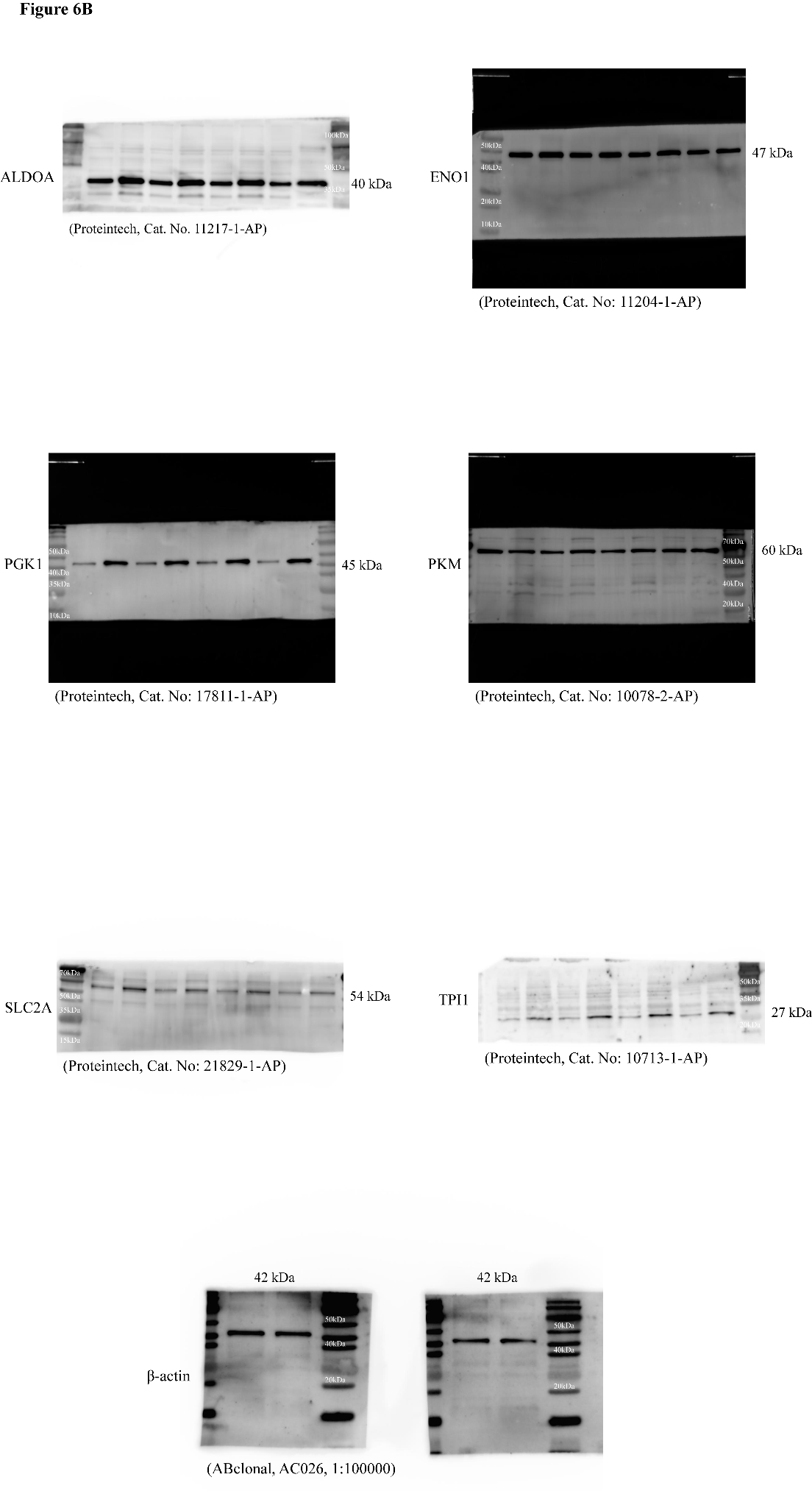
**
